# How the harm of drugs and their availability affect brain reactions to drug cues: a meta-analysis of 64 neuroimaging activation studies

**DOI:** 10.1038/s41398-020-01115-7

**Published:** 2020-12-14

**Authors:** F. Devoto, L. Zapparoli, G. Spinelli, G. Scotti, E. Paulesu

**Affiliations:** 1 Department of Psychology and PhD Program in Neuroscience of the School of Medicine and Surgery, University of Milano-Bicocca, Milan, Italy; 2 Department of Psychology, University of Milano-Bicocca, Milan, Italy; 3 fMRI Unit, IRCCS Orthopedic Institute Galeazzi, Milan, Italy

**Keywords:** Addiction, Human behaviour

## Abstract

Visual drug cues are powerful triggers of craving in drug abusers contributing to enduring addiction. According to previous qualitative reviews, the response of the orbitofrontal cortex to such cues is sensitive to whether subjects are seeking treatment. Here we re-evaluate this proposal and assessed whether the nature of the drug matters. To this end, we performed a quantitative meta-analysis of 64 neuroimaging studies on drug-cue reactivity across legal (nicotine, alcohol) or illegal substances (cocaine, heroin). We used the ALE algorithm and a hierarchical clustering analysis followed by a cluster composition statistical analysis to assess the association of brain clusters with the nature of the substance, treatment status, and their interaction. Visual drug cues activate the mesocorticolimbic system and more so in abusers of illegal substances, suggesting that the illegal substances considered induce a deeper sensitization of the reward circuitry. Treatment status had a different modulatory role for legal and illegal substance abusers in anterior cingulate and orbitofrontal areas involved in inter-temporal decision making. The class of the substance and the treatment status are crucial and interacting factors that modulate the neural reactivity to drug cues. The orbitofrontal cortex is not sensitive to the treatment status per se, rather to the interaction of these factors. We discuss that these varying effects might be mediated by internal predispositions such as the intention to quit from drugs and external contingencies such as the daily life environmental availability of the drugs, the ease of getting them and the time frame of potential reward through drug consumption.

## Introduction

Substance use disorder (SUD) is a chronically relapsing condition. Animal and human research has demonstrated that SUD, for either legal (alcohol, nicotine) and illegal (cocaine, heroin) substances, is associated with long-lasting neuroadaptations at the molecular, cellular, and circuitry level, that mediate the transition from goal-directed to habitual and compulsive drug intake^1,2^. Another crucial aspect of SUD is drug craving, defined as an intense desire for the substance. Drug craving can be triggered by the presence of the drug itself or drug-related stimuli, and it is accompanied by changes in physiological responses such as heart rate, sweating, and skin temperature^3^. As the enhanced response to drug-related cues may be a key factor contributing to the persistence of addiction^4^, the controlled exposure to the drug and drug-related stimuli (cue reactivity) has been widely used for the study of the physiological^5^ and neurofunctional^6,7^ correlates of drug craving. Recently, increasing efforts have been dedicated to the study of factors that can modulate the neural response to drug cues, such as addiction severity and drug availability or treatment status^8,9^. However, a systematic investigation of these effects in different populations with SUD is still lacking.

Below, after a brief overview of previous neuroimaging findings on the neural correlates of drug craving, we present a new meta-analytical study aimed at providing a quantitative assessment of how the nature of the substance of abuse and treatment status modulate the neural drug-cue reactivity.

### The neurobiology of craving: brain circuits mediating drug-cue reactivity

The exposure to drug-associated cues triggers motivational and emotional responses that influence decision making and the ensuing motor plans^10^. These are tightly linked to the nature of the substance, of its rewarding and reinforcing effects, as well as its availability. Neuroimaging studies^11–13^ and previous meta-analyses^14–16^ have shown that individuals with SUD exhibit altered neural responses in brain areas involved in different relevant aspects for craving.

People with SUD show altered activity in early visual cortices when exposed to drug-related cues vs. neutral objects^17^, presumably mediating the attentional bias towards the substance. They also exhibit increased activity in regions involved in incentive motivational processes^18,19^ of the mesocorticolimbic system^11,20,21^, in the ventral tegmental area (VTA) and its dopaminergic afferents to the ventral striatum, limbic structures (amygdala, hippocampus), and the prefrontal cortex (PFC). SUD is also associated with heightened responses in brain regions involved in the expression of habits^2^ and in processing knowledge about tool use^22,23^, such as the dorsal-striatal circuits and the inferior temporal, parietal, and motor cortices. This aberrant activity may favor drug-taking through the automatic activation of the semantic and motor representations associated with drug use^24^. Importantly, the activity in these regions in response to drug cues correlates with the severity of addiction for nicotine^12,25^, alcohol^26^, and cocaine^27^, and can predict relapse^28–30^.

Other brain structures may underlie higher-order cognitive processes such as reward expectancy, (the orbitofrontal cortex (OFC)), or action planning (dorsolateral prefrontal cortex (dlPFC))^31^. In their qualitative, yet thought-provoking, review, Wilson et al.^8^ discussed nineteen neuroimaging studies in individuals addicted to cocaine, heroin, alcohol, and nicotine: they proposed that activity in the PFC—in particular, in the dorsolateral and the orbitofrontal subdivisions—was typically seen in studies on not-seeking treatment patients (NST), rather than in studies on treatment-seeking patients (TS). Given the role of the OFC in integrating stimulus values^32^ and in representing the expected value of rewards^33^, and given the involvement of the dlPFC in planning and executing actions aimed at achieving the reward^34^, the authors proposed that frontal activity in NST reflects, at least in part, the expectation to obtain the drug after the experimental session^8^. The activity of these regions in response to cue-induced craving may thus underlie the evaluation of the availability of the substance, and the behavioral actions that are needed to pursue the goal.

A crucial, yet unanswered, question concerns the differences in the neural drug-cue reactivity across substances of different harm, legal or illegal^35^. These differ in several aspects: (i) they act through different molecular targets^36^, (ii) leading to different patterns of addiction and loss of behavioral control; (iii) they also differ in terms of overall availability and ease of being obtained. Finally, they can be administered through multiple routes, causing a different potency of the reinforcer^37^.

Here we tested the hypothesis that the way the brain becomes sensitized to drug-specific visual cues may depend on the nature of the substance of abuse (legal—tobacco, alcohol—or illegal—cocaine, heroin) and the desire of quitting from addiction and seeking treatment. Given the complex nature implied by such design [a 2×2×2 factorial design with four different groups of patients, two groups for each of two types of substances, and two kinds of visual stimuli (drug cues and control stimuli)], it would be beyond the strength of most to produce a study with this structure and sufficiently large samples on this matter. Moreover, to date, only a few studies have explicitly investigated the modulatory role of treatment status and/or drug availability on the neural reaction to drug cues^26,31,38,39^. However, when literature becomes sufficiently mature, meta-analyses may permit to test complex hypotheses that are normally out of reach. Here we present our attempt to achieve such a goal through a formal meta-analysis of previous imaging data considering 64 papers on the subject. The details of the rationale of our study and the methodologies employed are presented below.

### Aims and predictions

The goal of the current study was two-fold: first, to identify common and distinct neural correlates of craving triggered by visual anticipatory cues (we concentrated on anticipatory drug visual cues because experiments based on other sensory modalities (e.g., taste) are not sufficiently represented in the literature, nor they would permit to test the effects of interest over the entire spectrum of drugs, as the oral route is not the prevalent administration route for the illegal drugs considered, heroin or cocaine) across different populations of legal (alcohol, nicotine) and illegal (cocaine, heroin) substance abusers; second, to study the modulatory effect of treatment status on the neural drug-cue reactivity, per se and as a consequence of the type of substance.

We hypothesized that differences in the neural drug-cue reactivity patterns between the two classes of substances might reflect the different severity of addiction that they can induce^35^; in particular, we expected that the use of illegal substances would have been associated with stronger activation of brain regions involved in incentive salience and motivation, in line with the evidence that activity of these regions correlate with the severity of addiction and can predict relapse (see, for example, refs. ^28–30,40^). We further anticipated that treatment status could exert a modulatory effect in PFC responses^31,38,39^, according to the role of the OFC and dlPFC in encoding reward expectations and action planning in NST subjects^8^.

However, it remained a matter of empirical investigation whether such effects would be the same for legal and illegal substances: if not, this would suggest a complex interaction between biological and environmental factors not explicitly investigated so far in the imaging literature.

## Material and methods

### Data collection and preparation for meta-analysis

Records were retrieved through the following query in PubMed: “[cocaine OR heroin OR alcohol OR nicotine] AND [functional magnetic resonance imaging OR fMRI OR positron emission tomography OR PET OR neuroimaging] AND [addiction OR craving]”. The initial set of studies included 4240 papers, updated to March 2019.

Papers were included when fulfilling the following inclusion criteria:

- Populations involved: adult (mean group age ≥ 18 years) substance-dependent individuals according to the DSM-IV and DSM-5 criteria or similar, heavy drinkers^41^ or regular and abstinent drug users^42^; no minimum sample size was required; given the heterogeneity of abstinence time across studies, no consideration for the abstinence status was made;
- Anatomical conventions: we considered only data reported using MNI^43^ or Talairach^44^ coordinates exclusively from whole-brain analyses;
- Activation protocols: we considered only drug-cue reactivity paradigms based on both passive unimodal visual perception (with supraliminal stimuli) or mental imagery; this choice was motivated by our interest in anticipatory processing and by the need of curtailing the effect of potential confounds (e.g., sensory modality of stimulus presentation^9^);
- Statistical comparisons (linear contrasts) included: drug cue > control stimulus or baseline; data describing “deactivations” (drug cue < control stimulus) were not considered; only data from univariate analyses were considered (minimum threshold: *p* < 0.05 uncorrected); only contrasts related to simple effects of the group of substance-dependent individuals or interaction effects were included. For the interaction effects we considered only those testing a comparison like [drug cue > control]_SUD_ > [drug cue > control]_normal controls_;
- The regional effects were considered providing that they were measured from homogeneous populations (e.g., all treatment seekers);
- For studies assessing the effect of drug or drug treatments, we considered only studies that reported foci belonging to the pre-treatment and/or placebo condition^45^ or analyses corrected for treatment effects^46^.

See Supplementary Fig. S1 for the flowchart of the study search and selection process. The final data set included 64 studies, 90 statistical comparisons, and 1006 activation foci (see Supplementary Table S1 for further details on the studies included).

All the Talairach coordinates were converted to MNI space using the TAL2ICBM_SPM function^47,48^. Thirteen activation foci fell outside the less conservative mask of the GingerALE software (version 3.0.2^49,50^) and were excluded. The final data set comprised 993 foci, based on 1620 substance-dependent individuals (mean age: 36.9 years) with an average history of abuse of 11.56 years (information about the history of abuse was not available for 20 studies). Further details on the populations of the 64 studies are reported in the Supplementary Information (section 1.1).

### Hierarchical clustering analysis (HCA) and cluster composition analysis (CCA)

To identify anatomically coherent regional effects, we first performed an HCA using the unique solution clustering algorithm^51^ implemented in the software CluB^48^, as described in previous meta-analyses^52–54^ and in a specific methodological paper^48^. The spatial resolution of our analyses was set to be 5 mm. In short, the method implies finding an optimal clustering solution that is then followed by a cluster composition statistical analysis (CCA). The HCA takes into account the squared Euclidian distance between each pair of foci included in the data set. The clusters with minimal dissimilarity are recursively merged using Ward’s criterion^55^, to minimize the intra-cluster variability and maximizing the between-cluster sum of squares^51^. The CCA allowed us to test the statistical association of each cluster with the factors of interest. For the CCA, each focus of activation was classified according to two factors of interest: (i) class of substances (*legal* vs. *illegal*) and (ii) treatment status of the participants (*treatment-seeking* (TS) vs *not-seeking treatment* (NST)) (see section 1.2 in the Supplementary Information for further details on the data collection and foci classification process). This implied calculation for each cluster of the proportion of foci belonging to different levels of each factor. Such proportion was then compared with a target proportion reflecting the overall distribution of foci classified according to our factors of interest in the whole data set (prior likelihood, PL).

Main effects for the factors Class of substances and Treatment status were assessed using binomial tests on the proportion of foci associated with each level of the two factors within each cluster. To test the Class of substances-by-Treatment Status interactions, we used the Fisher’s Exact test^56^ on the empirical peak distribution within each cluster (weighted on the PL). To interpret the direction of the significant interaction effects, we calculated the ratio between the proportion of observed foci and the total number of foci within the cluster (observed probability, OP)^52^. Then, we divided this value for the PL: this computation (OP/PL) results in an index that indicates the degree to which the distribution of activation peaks belonging to a specific combination of factors within a cluster exceeds the expected probability. Values greater than one indicate a higher probability for the cluster to be specific for that combination of factors.

We considered for further discussion only clusters with at least four contributing studies (equal or greater than the 25th percentile of the total contributing studies); moreover, we discarded those clusters with cardinality (the number of peaks) inferior to the 25th percentile (<5) of the total cardinality.

Clusters whose one-tailed *p*-value was greater than or equal to 0.5 for both levels of the factor Class of substances were considered as of high chance of being genuinely undifferentiated^57^.

### Validation of the spatial relevance of each cluster using the ALE procedure

To validate the results of the HCA, we assessed the spatial significance of the HCA solutions by comparison with a standard activation likelihood estimate meta-analysis of the same raw-data. Here we used the Turkeltaub non-additive method^50^, with the cluster-forming statistical threshold of *p* < 0.05 FWE-corrected and corrections for contrasts coming from the same study^50,58^. Only clusters surviving the spatial intersection between the HCA and ALE maps, were then taken into account for additional analyses and discussion.

### Further methods of interpretation of the results

Besides typical forward inferences based on the experimental design and interaction of factors, we also relied on quantitative reverse inference when needed. Each CCA map representing the effect of the factor of interest was loaded into the Neurosynth database and analyzed by means of the “decoder” function (http://neurosynth.org/decode/). The decoder function of Neurosynth allows one to retrieve the Pearson correlation of the keywords that are most associated with the input image, containing the clusters identified by the meta-analysis, based on the NeuroVault repository. The *r*-value associated with each keyword reflects the correlation across all voxels between the input map and the map associated with a particular keyword in NeuroVault. We considered the first 15 words associated with each CCA map, after excluding anatomy-related terms and duplicate terms. The Neurosynth analysis followed our best interpretation in preliminary writing of the discussion: its results are mentioned by the end of each section of the discussion.

## Results

### Hierarchical clustering analysis and cluster composition analysis

The HCA identified 117 clusters, each composed of 2–24 peaks; the mean standard deviation along the three axes was 4.96 mm (*x* axis), 4.93 mm (*y* axis) and 4.92 mm (*z* axis). Thirty-five clusters were retained following the intersection procedure with the ALE map (the results of the ALE analysis are reported in Supplementary Fig. S2). One cluster was excluded because its cardinality fell below the 25th percentile of the total cardinality of the clusters (cardinality < 5 foci).

On average, these clusters contained 13 foci (range: 5–24), with 3–17 studies (mean: 8) contributing to each cluster. The full list of clusters overlapping with the ALE maps is available in the Supplementary Information (Supplementary Table S2). The results of the CCA are reported in Table 1, whereas the full list of terms identified by Neurosynth for each CCA map is reported in Supplementary Table S3.

**Table 1:** Results of the cluster composition analysis.

		Left hemisphere			Right hemisphere					Class of substances		Treatment status		Interaction
Cluster ID	Centroid label (BA)	*X* (SD)	*Y* (SD)	*Z* (SD)	*X* (SD)	*Y* (SD)	*Z* (SD)	# of foci	# of contributing studies	Legal	Illegal	Treatment seeking	Not-seeking treatment	Class of substances-by-treatment status
21	Inferior occipital gyrus (19)	−46 (4.5)	−70 (3.8)	−8 (3.9)				17	4	0.616^a^	0.583^a^	0.552	0.645	0.102
22	Inferior temporal gyrus (37)	−51 (3.4)	−63 (4.2)	−5 (5.8)				17	8	0.995	0.027^b^	0.076	0.977	1
28	Caudate nucleus	−13 (6.7)	17 (3.8)	2 (4)				9	7	0.687	0.582	0.794	0.441	0.032^b^
30	Nucleus accumbens				8 (3.4)	8 (5.6)	−6 (6.1)	19	10	0.986	0.049^b^	0.597	0.59	1
47	Middle occipital gyrus (19)				49 (3.7)	−77 (4.9)	4 (4.3)	6	5	0.7^a^	0.626^a^	0.607	0.716	1
62	Calcarine scissure/precuneus (17/30)	0 (6.3)	−59 (5.1)	15 (4)				12	6	0.995	0.036^b^	0.032^b^	0.996	1
68	Lingual gyrus (27)	−15 (5.3)	−35 (4.8)	−6 (5.3)				10	9	0.515^a^	0.728^a^	0.461	0.779	1
69	Midbrain (ventral tegmental area)	−5 (2.8)	−23 (6.7)	−9 (4.7)				12	9	0.995	0.036^b^	0.032^b^	0.996	0.438
92	Medial orbitofrontal gyrus (10)	−5 (5.6)	52 (4.7)	−4 (4.9)				15	11	0.47	0.726	0.701	0.5	0.006^c^
96	Supragenual anterior cingulate cortex (32)	−5 (5.9)	45 (4.5)	18 (5.8)				20	9	0.622^a^	0.562^a^	0.087	0.97	0.54
97	Hippocampus/amygdala (35/28)	−21 (4.2)	−5 (3.7)	−21 (3.3)				17	9	1	0.005^c^	0.076	0.977	1
103	Perigenual anterior cingulate cortex (32)				2 (4.5)	40 (3.5)	5 (7)	16	12	0.344	0.817	0.908	0.204	0.045^b^
114	Dorsal anterior cingulate cortex (24)	0 (2.3)	29 (4.2)	22 (6.1)				13	7	0.003^c^	1	0.999	0.004^c^	1
116	Thalamus				1 (3.6)	−11 (3.7)	4 (4.7)	24	10	0.913	0.181	0.158	0.927	0.04^b^

For each cluster, the following information is reported: cluster ID, anatomical label according to the AAL (and Brodmann area, BA), centroid coordinates (standard deviation) in MNI, number of contributing foci, number of contributing studies, *p*-values for the binomial tests (class of substances, treatment status) and for the Fisher’s exact tests (class of substances-by-treatment status interactions).

^a^Undifferentiated clusters.

^b^*p* < 0.05.

^c^*p* < 0.01.

### Undifferentiated clusters

There were undifferentiated clusters, that is spatially significant clusters, consistently activated in the basic drug-cue paradigm, yet with no association with a specific drug class: these were located in the left lingual gyrus, left anterior cingulate cortex (ACC), left inferior occipital gyrus, and right middle occipital gyrus (Supplementary Fig. S3). The 15 terms with the higher *r*-values, according to Neurosynth, are reported in Supplementary Fig. S3 caption; the top five were *traits (personality), mentalizing, beliefs, craving, visual stimuli*.

### Effect of class of substances

**Fig. 1:**
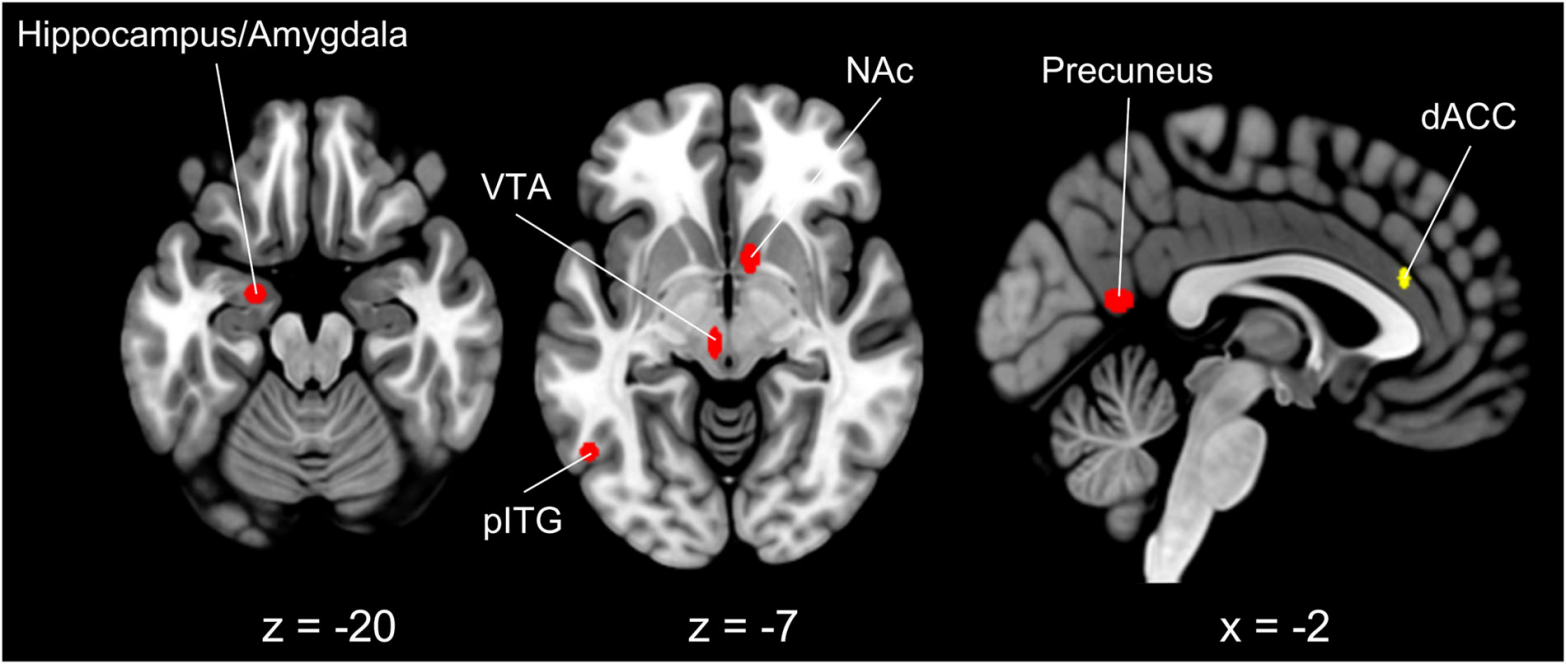
Results of the binomial CCA for the factor “class of substances”. Clusters more frequently activated by individuals addicted to legal substances are depicted in yellow, whereas clusters more frequently activated by individuals addicted to illegal substances are depicted in red. Slice coordinates are reported in MNI stereotaxic space. The decoder function of Neurosynth returned the following 15 terms with the highest association with the CCA map (decreasing order): tools, motivational, anticipation, addiction, reward anticipation, outcome, subjective, behavior, monetary reward, complex, probabilistic, objects, incentive delay, form, sighted. dACC dorsal anterior cingulate cortex, pITG posterior inferior temporal gyrus, VTA ventral tegmental area.

Legal substance abusers showed more frequent activity of the medial dorsal anterior cingulate cortex (dACC) (Fig. 1, in yellow). Illegal substance abusers showed more frequent activity in the left posterior inferior temporal gyrus (pITG), anterior hippocampus/amygdala, in the medial calcarine cortex and precuneus, the right caudate/nucleus accumbens, and the left midbrain (VTA) (Fig. 1, in red). The 15 Neurosynth terms with the higher *r*-values are reported in Fig. 1 caption; the top five were: *tools, motivational, anticipation, addiction, reward anticipation*.

### Effect of treatment status

**Fig. 2:**
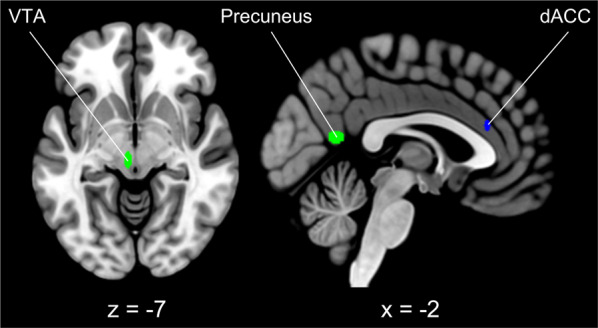
Results of the binomial CCA for the factor “treatment status”. Clusters specific for treatment-seeking individuals are depicted in green, whereas the cluster specific for not-seeking treatment subjects is depicted in blue. The decoder function of Neurosynth returned the following 15 terms with the highest association with the CCA map (decreasing order): aversive, reversal (learning), anticipatory, heart (rate), intense (emotion), episodic memory, autobiographical, cognitive emotional, force, fear, reward, mild cognitive, pain, personal, sensation. Slice coordinates are reported in MNI stereotaxic space. dACC dorsal anterior cingulate cortex.

TS patients activated more frequently the calcarine cortex and the precuneus and the midbrain, in a region compatible with the ventral tegmental area (VTA) (Fig. 2, in green), whereas NST patients activated more frequently the medial dACC (Fig. 2, in blue). The 15 terms with the higher *r*-values are reported in Fig. 2 caption; the top five were: *aversive, reversal (learning), anticipatory, heart (rate), intense (emotion)*.

### Class of substances-by-treatment status interactions

**Fig. 3:**
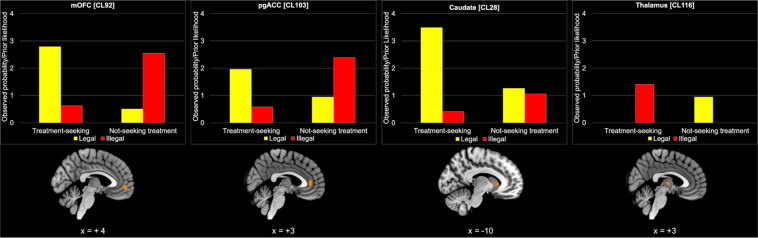
Results of Fisher’s Exact test for the “class of substances”-by-“treatment status” interactions. Clusters with a significant class of substances-by-treatment status interaction are shown in orange, along with their respective plot. On the *y* axis of the bar-plots is represented the ratio between the observed probability and the prior likelihood: the more this value exceeds 1, the more the cluster is associated with that combination of factors. The decoder function of Neurosynth returned the following 15 terms with the highest association with the CCA map (decreasing order): engagement, referential (self), value, reward, traits (personality), choose, arousal, task positive, autobiographical, monetary, moral (decision making), contexts, monetary incentive, expectations, valence. Slice coordinates are reported in MNI stereotaxic space. pgACC perigenual anterior cingulate cortex, mOFG medial orbitofrontal gyrus.

The Fisher’s Exact test revealed four clusters with a significant interaction between the class of substances and treatment status of the participants. These clusters were located in the left medial orbitofrontal gyrus (mOFC), in the right perigenual ACC (pgACC), in the right thalamus, and in the left caudate nucleus (Fig. 3).

The interaction plots presented in Fig. 3 show that the mOFC and the ACC were more frequently activated by TS subjects addicted to legal substances, and by NST individuals addicted to illegal substances.

On the other hand, the caudate nucleus was more frequently activated by TS compared with NST individuals, specifically for legal substances. On the contrary, the right thalamus was associated with TS compared with NST subjects, particularly for individuals addicted to illegal substances. The 15 terms with the higher *r*-values are reported in Fig. 3 caption; the top five were: *engagement, referential (self), value, reward, traits (personality)*.

## Discussion

Legal and illegal drugs differ in several respects. Alcohol and tobacco/nicotine are freely available in the environment: they can be found 24/7 in shops at a low-to-moderate monetary cost. Conversely, illegal drugs like cocaine and heroin are less widely available, they are associated with a severe degree of harm and dependence^35^, they are usually sold at very high prices per unit weight in the illegal market^59^, and their trading implies the risks associated with a criminal action. The present meta-analysis of neuroimaging studies on drug-cue reactivity, for the first time, assessed whether, and how, the nature of these drugs and treatment status can interact at the neurobiological level, giving rise to specific brain activation patterns in response to drug cues.

### Common neural correlates of craving across legal and illegal substances

The sight of drug-related cues, compared with neutral stimuli, activates occipital cortices and anterior cingulate cortex in both legal and illegal drug consumers: this is consistent with the perceptual salience of drug-related stimuli (see section 2.1. in the Supplementary Information for a supplementary discussion). To our surprise, the Neurosynth classification algorithm went beyond this simple interpretation adding terms like *traits*, *mentalizing, beliefs, craving*, much in line with the origin of the data to which the algorithm was blind at the time of its interrogation.

### Distinct neural correlates of drug-cue reactivity for legal and illegal substances

Consistent with the fact that cocaine or heroin can be severely addictive inducing extreme craving, we found a more frequent activation of the subcortical reward pathway (the VTA, NAc, the amygdala) in illegal drug abusers. This evidence is also in agreement with a large body of animal and human studies suggesting that aberrant activity of the mesocorticolimbic pathway may be responsible for this phenomenon: the VTA, the NAc and the amygdala are crucial structures for the expression of cue-elicited reward-seeking behaviors^18,60^. In humans, the activity of the ventral striatum (which includes the NAc) during cue reactivity predicts relapse in heroin^40^ and alcohol-dependent individuals^61^, and NAc resting-state functional connectivity with the dlPFC predicts relapse in cocaine-dependent individuals^62^. These findings also align with the evidence that measures of addiction severity correlate with cue-induced activity in these regions^25–27^.

Unexpectedly, two other brain regions outside the mesocorticolimbic system were more frequently activated in addicted to illegal substances, the inferior temporal cortex, and the precuneus.

The precuneus is part of the default mode network: all the well-known associated behavioral dimensions may apply^63^, including enhanced attentional anticipation for external stimuli^64,65^.

The inferior temporal cortex is part of a network that stores and processes knowledge about object manipulation and tool use^23,24^. Its involvement may reflect automatic bottom-up phenomena representing, in a broad sense, the “affordances” for the particular substance of abuse. Such bottom-up phenomena would be stronger the more severe the condition of abuse^9^.

Interestingly, the only brain region that was more frequently activated in legal substance abusers was the dACC. In nicotine addiction, the dACC activity was tentatively associated with the effort of directing the attention away from the stimulus to suppress the craving, as immediate consumption was impossible^15,16^: indeed, cognitive control over craving may be especially required when the possibility of consuming the substance is a more concrete one, as in the case of legal substances, alcohol, and nicotine. All these interpretations were broadly confirmed by the quantitative semantic associations made by Neurosynth.

### The effect of treatment status in legal and illegal substances

Contrary to what one could have predicted according to the previous investigations^8,31^, the brain activation patterns of TS and NST differed only for a small number of regions, all outside prefrontal cortex: these included the VTA and the precuneus (associated with TS and illegal substances) and the dACC (associated with NST and legal substances). Exploration of the *Neurosynth* database showed that the clusters associated with treatment status, as the main effect, are linked to cognitive-emotional processes and reward anticipation. Of great interest is the association of these regions also with reverse learning paradigms: one can imagine that seeing a drug cue in a treatment-seeking status may trigger processes needed to change the associated value with the cue and the actions typically involved.

On the other hand, we found two cortical regions where the nature of the substance of abuse and the treatment status of a participant did interact: the perigenual ACC (pgACC) and the medial OFC (mOFC). More specifically, these two regions were more frequently activated in NST consumers of illegal substances and in TS consumers of legal substances. Interestingly, these include the same cortical region, the OFC, that according to Wilson et al.^8^ should display an association with NST. Our data show that this is not the case and that its association with treatment status is modulated by the nature of the drug (see section 2.2 in the Supplementary Information for a further discussion about the differences with the observations of Wilson et al.^8^).

The medial and ventral portion of the OFC (also called ventromedial PFC), together with the ACC, is part of a network that mediates inter-temporal decision making, as suggested by neuroimaging meta-analyses on temporal discounting phenomena (these are tested in experimental situations in which an individual is forced to choose between a later—but larger —or an earlier—but smaller—reward^66,67^) and by clinical evidence on patients with OFC lesions, whose decisions are characterized by the insensitivity to future consequences and by the preference for immediate reward^68^. Further, OFC (in particular, Brodmann area 10) activity correlates with the ease and difficulty of the choice^69^, and it is functionally connected with the pgACC^70^, a region that is thought to represent action-reward associations^71^. The interpretations of the role of these regions in temporal discounting (keyword: *expectations*, *value*) and decision making (keywords: *choose, moral (decision-making)*) is also consistent with a Neurosynth analysis.

In keeping with the above interpretation of our data, we acknowledge that the direction of the interactions observed here may be driven by a number of factors that reflect the intrinsic differences between classes of substances and treatment types. First, it is worth recalling that illegal substances, here heroin and cocaine, are associated with more profound brain reactions to drug cues in general and they are, by definition, less widely available or affordable, if compared with legal substances, here alcohol and tobacco. Second, the availability of substances of abuse differs depending on the status of treatment seeking and the nature of the substance: for example, a treatment seeker, abuser of illegal substances, is frequently an inpatient submitted to a forced regimen of withdrawal from the drug (of the studies reviewed here, at least 10 out of 23 studies involved inpatients for the illegal drugs groups). Third, getting illegal substances exposes to the risk of dealing with crime, often leading to enforced treatment, while legal substances can be obtained without such risk.

**Fig. 4:**
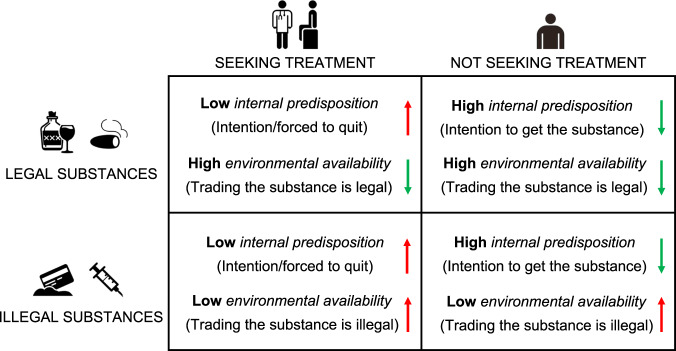
Internal and environmental determinants in drug-cue reactivity. A simplified model of the interaction between class of substances and treatment status in the PFC. More frequent activity in regions involved in inter-temporal decision making and reward evaluation may reflect the incongruency between determinants that signal shorter delay/immediate reward (downward green arrows) vs. longer delay/absence of reward (upward red arrows): when these contingencies signal conflicting information about the potential time frame of the reward (represented by the arrows pointing in different directions), additional decision making, and reward evaluation processes may be required to form an expectation about the delay of drug consumption. The interaction effect observed here may be in part mediated by the different drug availability implied by the type of treatment usually considered for legal (outpatient—getting the substance soon is still possible) vs. illegal (inpatient—getting the substance soon is less likely) substance abusers.

Following these considerations, one may argue that the interaction effects seen in regions concerned with the representation of reward value and decision making (here, the pgACC and the mOFC) may reflect conflictual situations in which internal predispositions and drug availability clash when subjects are exposed to drug cues: as illustrated in Fig. 4, this is exactly what may happen when one is determined to quit and/or under treatment and yet he is exposed to an easily available drug (legal drugs here) or when one is not determined to quit and at the mercy of limited availability (in quantity and/or price) typical of the illegal drug market. The way time is represented in these brain regions, with respect to reward and the variable delay whereby this is gained, may be an important factor here. For example, for a cigarette smoker seeking for treatment (low internal predisposition to consumption and high environmental availability) or for a crack-cocaine abuser not seeking for treatment (high internal predisposition to consumption and low environmental availability), a drug cue represents (i) a substance that should be not consumed soon—the patient is under treatment—but that is highly available in the environment or (ii) a substance that one may want to consume soon—the patient is not under treatment—but that is costly and poorly available in the environment, thus making the outcome less predictable (Fig. 4).

As a consequence, the interaction effects seen for a class of substances and treatment status in the pgACC and mOFC may reflect the recruitment of additional reward evaluation and decision-making processes, which are required to form, and stressed by, the expectations about the potential delay of drug consumption after exposure to drug cues.

Unexpectedly, two subcortical structures—whose activity is usually not observed in studies where treatment status/drug availability is explicitly manipulated^31,38,39^—showed a significant interaction between a class of substances and treatment status: the caudate nucleus and the thalamus. In particular, both regions were more frequently activated by TS participants, but the caudate nucleus was more frequently activated by legal substance abusers, the thalamus by illegal substance abusers.

The caudate nucleus is functionally and structurally connected to multiple brain areas involved in emotion, cognition, and action^72^, and it supports efficient goal-directed actions through the selection of appropriate behavioral schemata^73^. Goal-oriented vs. habitual behavior is indeed crucial for those individuals that are seeking treatment, not for those NST. Conversely, activity of the thalamus has been associated with drug craving and with addiction severity in previous animal and human neuroimaging studies^26,74^, even if its precise contribution to the experience of drug craving is still unclear. If anything, our results show that subcortical structures such as the caudate nucleus and the thalamus are modulated by some aspects of treatment status and/or drug availability that are specific for a particular class of substances (see section 2.3 in the Supplementary Information for a discussion of the strengths and limitations of our approach).

## Conclusions and implications for clinical sciences

Taken together, these findings may suggest some initial practical considerations: drug-cue brain reactivity, an index of craving intensity and, possibly, of the risk of relapse into addiction, is not only influenced by the potential harm of a given substance but rather it also depends from internal and contextual determinants. As treatment-seeking patients are characterized by the engagement of specific brain reactions to drug cues depending on the substance of abuse, rehabilitation, particularly when cue-extinction strategies are employed^75^, may thus benefit from tailor-made interventions that consider the influence of internal and environmental contingencies when subjects are likely to be exposed to drug cues.

## Supplementary information

Supplemental Information

## Data Availability

The entire algorithm and the MATLAB script associated with the software CluB are available at the webpage: https://bit.ly/CLU_B.
