# Supplementary material for: How the harm of drugs and their availability affect brain reactions to drug cues: a meta-analysis of 64 neuroimaging activation studies
Source: Transl Psychiatry. 2020 Dec 14;10:429. doi: 10.1038/s41398-020-01115-7 (PMC7736294; doi:10.1038/s41398-020-01115-7)
Supplement: Supplementary file 1 — Supplemental Information [file 41398_2020_1115_MOESM1_ESM.docx]

**How the harm of drugs and their availability affect brain reactions to drug cues: a meta-analysis of 64 neuroimaging activation studies.**

Devoto F.^1^, Zapparoli L.^2^, Spinelli G.^2^, Scotti G.^2^ and E. Paulesu^2,3^

1. Department of Psychology and PhD Program in Neuroscience of the School of Medicine and Surgery, University of Milano-Bicocca, Milan, Italy
2. Department of Psychology, University of Milano-Bicocca, Milan, Italy
3. IRCCS Orthopedic Institute Galeazzi, fMRI Unit, Milan, Italy

***Supplementary Materials***

1. Supplementary Methods

1.1. Sample characteristics of the included studies 2

1.2. Data collection and foci classification 3

2. Supplementary Discussion

2.1. Common neural correlates of craving across legal and illegal substances 4

2.2. Likely causes of the differences with the observations of Wilson et al. (2004) 5

2.3. Strengths and limitations 5

3. Supplementary Tables

3.1. Table S1 8

3.2. Table S2 26

3.3. Table S3 29

4. Supplementary Figures

4.1. Figure S1 34

4.2. Figure S2 35

4.3. Figure S3 36

5. Supplementary References

5.1. References 37

**1. Supplementary methods**

**1.1. Sample characteristics of the included studies**

In the end, the final dataset was based on 1558 substance-dependent individuals (mean age: 36.9 years) with an average history of abuse of 11.56 years (information about the history of abuse was not available for 20 studies). In the majority of the studies, the sample included only males (24 studies, 37.5 %), or the majority of male participants (25 studies, 39.1%), whereas in the minority of the studies the sample included only females (3 studies, 4.7%) or the majority of female participants (1 study, 1.6%). In 9 studies (14.1%) gender of the participants was balanced, whereas in 2 studies (3%) this information was not available. Studies on legal (M = 22, SD = 17) and illegal substances (M = 23, SD = 10), on average, **did not differ significantly with respect to sample size** (Wilcoxon test, W = 448.5, p = .12). TS (M = 25, SD = 16) and NST individuals (M = 21, SD = 12), on average, **did not differ significantly with respect to sample size** (Wilcoxon test, W = 502.5, p = .71).

Subjects from studies on legal (M = 35.4 years, SD = 8.6 years, NA = 1 study) and illegal substances (M = 38.5 years, SD = 6.2 years, NA = 0 studies), on average, **did not differ significantly with respect to age** (Wilcoxon test, W = 630, p = .07). TS (M = 36.3 years, SD = 5.9 years, NA = 0 studies) and NST individuals (M = 37.8 years, SD = 9.3 years, NA = 1 study) **did not differ significantly in terms of mean age** of the participants (Wilcoxon test, W = 472, p = .81). Studies on legal (M = 13.2 years, SD = 7.4 years, NA = 15 studies) and illegal substances (M = 10.8 years, SD = 4.9 years, NA = 5 studies) **did not differ significantly in terms of history of abuse** (Wilcoxon test, W = 235.5, p = .29), whereas studies with **treatment-seeking participants on average had a briefer history of abuse** (M = 10.6 years, SD = 4.6 years, NA = 8 studies) compared to not-seeking treatment participants (M = 15 years, SD = 7.2 years, NA = 12 studies; Wilcoxon test, W = 129, p = .03).

**1.2. Data collection and foci classification**

The final dataset included 64 studies (cocaine = 19**^[[1]](#footnote-1)^**, heroin = 13, alcohol = 16, nicotine = 16), 90 statistical comparisons (experiments in the GingerALE jargon^1,2^) (cocaine = 24, heroin = 25, alcohol = 19, nicotine = 22) and 993 activation foci (see Figure S1 and Table S1 for further details on the studies included).

For the Cluster Composition Analysis, each focus of activation was classified according to two factors of interest: (i) **class of substances** (*legal* vs. *illegal*) and (ii) **treatment status** of the participants (*treatment-seeking* (TS) vs *not-seeking treatment* (NST)).

In particular, foci coming from studies with individuals addicted to alcohol or nicotine were classified as *legal,* whereas foci from studies with individuals addicted to cocaine or heroin were labeled as *illegal*. Accordingly, this labeling encoded a between-group factor that we call **class of substances**. Activation foci were also classified according to the **treatment status** of the participants: only the activation foci belonging to studies with treatment-seeking participants as specified in the manuscript^3,4^, with participants involved in clinical ­trials^5-7^ or admitted/to-be-admitted to inpatient^8,9^ or outpatient treatment^10,11^ or recruited from drug services or rehabilitation centers^12,13^ were classified as *treatment-seeking* (TS – participants whose drug use is limited by the willingness to quit and/or by the restricted access to the substance); by contrast, activation foci generated by participants that are not involved in any treatment nor clinical^14^ or that are active users recruited from the community^15,16^ were classified as *not-seeking treatment* (NST – participants that are not motivated to quit and whose access to the substance is not restricted).

**2. Supplementary discussion**

**2.1. Common neural correlates of craving across legal and illegal substances**

We found that a fronto-occipital network, comprising three clusters located in the occipital cortex (IOG, MOG, lingual gyrus) and one in the prefrontal cortex, in the ACC, was activated in response to drug cues regardless of the type of substance, suggesting the existence of a core brain circuit underlying cue-induced drug craving.

Occipital cortex activity in response to drug-cues has been consistently reported in previous meta-analyses of neuroimaging studies^17-19^, and it has been associated with craving^20^ and meta-cognitive processes, such as awareness of problematic drug use^21^, in previous experimental studies. In a recent ALE meta-analysis, Hanlon and colleagues demonstrated the replicability of the occipital cortex finding (Brodmann areas 19 and 17) across legal and illegal substance abusers, suggesting that occipital activity may reflect the attentional bias towards drug-related stimuli or the rewarding properties of the substance per se^22^. If one accepts that the legal and illegal substances included here vary with respect to reinforcing properties^23^ and to the degree of dependence induced^24^, our finding does not support the idea that occipital cortex activity reflects the reinforcing properties of the substance itself: indeed, they are more compatible with the hypothesis that occipital cortex activity reflects bottom-up attentional phenomena towards drug-associated stimuli, a process that is shared across abusers of different substances; similar findings have been observed for visual stimuli evoking other rewarding contingencies, like erotic and sexual images^25,26^.

Another cluster, located in the anterior cingulate cortex (ACC), was undifferentiated with respect to the class of substances. Activity of the ACC in response to drug cues has been previously associated with reinforcement-guided decision-making (i.e., integration of information including previous experiences with the reward)^27^, cognitive control^18,19^, and reward value representation^28^. Further, based on the evidence that intact glutamatergic projections from the PFC – which includes the ACC – to the NAc and the ventral pallidum are necessary to trigger drug-seeking in animal models^29,30^, this network has been considered as a “final common pathway” mediating the initiation of drug-seeking^31^. Relevant to this interpretation, a qualitative analysis of the terms identified by Neurosynth is consistent with the idea that this pattern of activation reflects the deployment of attention to rewarding visual stimuli and, ultimately, to craving.

**2.2. Likely causes of the differences with the observations of Wilson et al. (2004)**

Contrary to what described by Wilson et al. (2004), a systematic association with prefrontal cortex activation and the not-seeking treatment status, we did not find such main effect, neither in dorsolateral prefrontal cortex, nor in orbitofrontal cortex. We rather found that this association was dependent on the nature of the substance of abuse in orbitofrontal cortex. While it was impossible to replicate with a coordinate based meta-analysis Wilson's et al. (2004) results because 8 of the studies that they considered were based on ROIs analyses, there are further analytical differences to prevent a formal comparison. First, Wilson et al. (2004) used a nominal analysis of the activation patterns as broad as the aforementioned terms (dlPFC; OFC); we used a coordinate-based meta-analysis with 5 mm spatial resolution and a ALE correction for spatial extent; we made a statistical assessment of the relevance of the clusters identified. In addition, Wilson et al. (2004) did not make a statistical evaluation of their findings. In retrospect, a chi-squared analysis based on their Table 1 would be significant for the dlPFC but not for the OFC. Further, Wilson's et al (2004) is based on nineteen studies while we considered sixty-four studies. All these differences may have contributed to our inability to replicate Wilson's et. al (2004) claim.

**2.3. Strengths and limitations**

Along with its limitations, our study has also several strengths. First, we explored for the first time the interaction effects between the class of substances and treatment status^32,33^, which represents a step forward in the understanding of the neurobiology of drug craving. Second, we selected a highly homogeneous set of imaging studies on the cue-reactivity paradigm (in either visual or imagery modality) while preserving a moderate sample size (64 studies included), which constitutes another strength of our study. Third, we combined the ALE method^1^, which reveals the brain regions with most convergent activation across the whole dataset, with hierarchical clustering and post-hoc statistical characterization of the clusters concerning the factors of interest^34^: this approach has been validated in other domains of cognitive neuroscience, such as in the domain of food perception and obesity^35^, human volition^36^, reading dyslexia^37^, single-word reading^38^, and noun and verb processing^39^.

However, we acknowledge that our work has some limitations too. A first obvious limitation concerns the differences in demographic characteristics (e.g., sex distribution, co-occurring disorders, socio-economic status) of the samples across class of substances and treatment status; yet, we acknowledge that this becomes an issue if there is a systematic association of these nuisance variable with one level of our factorial design. If not, one has to assume that the emerging random noise would cancel out. Another limitation concerns the cross-sectional nature of the studies included, which means that we cannot exclude that some of our results reflect brain activation patterns pre-existent to the SUD. Also, we cannot exclude that some of the effects observed in our meta-analysis are due to abstinence-related effects, since abstinence has can potentiate the reward responses to drug cues^8,13,40,41^; due to the intrinsic difference in average abstinence across substances, it was not possible to include this factor in our analysis. Similarly, the length of drug use has been shown to exert a modulatory effect on the neural response to drug cues^33^, but we could not take this factor into account due to the high number of studies that did not report this information. Nonetheless, as studies with treatment-seeking individuals have on average a shorter history of abuse compared to studies with not-seeking treatment individuals, we cannot assess the degree to which the observed effects of treatment status are mediated by the history of drug use. Another source of confound may concern the pharmacological effects of methadone-assisted heroin-addicted individuals, as in some of the included studies on heroin addiction, subjects were undergoing methadone-assisted detoxification or were under methadone treatment^10,12,13,42,43^. In a similar fashion, it is hard or even impossible to isolate treatment type (inpatient vs outpatient, voluntary vs forced) from the effect of class of substances (legal vs illegal), as illegal drugs abusers, due to the illegal nature of the substance, are more likely to be enrolled in forced and in-patient treatments compared to legal drug abusers (e.g., no forced in-patient treatments are expected for nicotine-dependent individuals). However, we acknowledge that these limitations reflect the clinical complexity of the matter at stake when considering studies on human subjects, and that the influence of these nuisance variables becomes an issue only when there is a strong and systematic association with one level of our factorial design. If not, one has to assume that the emerging random noise would cancel out.

Consistent with the real-world clinical complexity of the human SUD, in our pool of selected studies, some participants were poly-substance abusers, and most of them had coexisting nicotine addiction, thus raising the possibility that poly-substance use may have affected our results. Also, based on the fact that the potency of a reinforcer can be estimated from its main route of administration^23^, we cannot exclude that inter-study differences in the route of administration within- and between-substance may have affected our results: again, this was impossible to account for, given the multiple ways in which a single substance can be administered, and because many studies did not report this information. The latter issue is indeed crucial, as the effect of the route of administration on the neural responses to drug cues (in individuals addicted to the same substance) would point to a new relevant factor worth of investigation.

**3. Supplementary tables**

**3.1. Table S1**

| **Author(s)** | **Drug of Primary Abuse** | **Cue-reactivity Paradigm** | **Sample size** | **Sample characteristics** | **Drug cue** | **Baseline condition** | **Cue-induced craving assessment** | **Abstinence** | **Main route of administration** | **History of abuse** | **Additional substances** | **Treatment status** |
| --- | --- | --- | --- | --- | --- | --- | --- | --- | --- | --- | --- | --- |
| Bach et al., 2019^9^ | **Alcohol** | - ***Imaging:*** fMRI.  - ***Modality:*** Visual (Pictures).  - ***Design:*** Block.  Participants were instructed to watch and attend to the stimuli. | **DA**: 50  **HC**: 35 | **Sex**  *DA:* all men HC: all men  **Co-occurring disorders**  No other axis I psychiatric condition other than alcohol abuse or dependence, except nicotine dependence  **Socio-economic status**  *DA- education*   - 2 post-secondary education - 14 apprenticeship only - 32 attended college or higher   *HC- education*   - 0 post-secondary education - 5 apprenticeship only - 30 attended college or higher | ***Alcohol:*** Alcohol-related pictures (wine, beer, spirit). | ***Neutral:*** Neutral objects. | No assessment of cue-induced craving. | 17.2 days (averaged between the groups) | Ingestion | 17.56 years (averaged between the groups) | Cigarettes | TS (in-patient treatment) |
| Cortese et al., 2015^44^ | **Nicotine** | - ***Imaging:*** fMRI.  - ***Modality:*** Visual (Pictures).  - ***Design:*** Block.  Participants were instructed to watch and attend to the stimuli. | ***DA***:17 | **Sex**  *DA:* 13 men, 4 women  **Co-occurring disorders**  No other axis I psychiatric condition other than nicotine and caffeine abuse or dependence  **Socio-economic status**  Not reported | ***Nicotine:*** Smoking-related pictures (advertisement or digital photos). | ***Neutral:*** Neutral images matched for intensity, color and complexity. | ***Assessment:*** Before fMRI.  ***Scale:*** Three 11-point Likert scales to measure craving amount [0 = ‘very little’; 10 = ‘a great deal], craving intensity [0 = ‘none’, 10 = ‘irresistible’] and craving control [0 = ‘no control’, 10 = ‘complete control’].  ***Results:*** Amount: mean = 7.6 (2.6); Intensity: mean = 6.5 (2.7); Control: mean = 6.5 (3.1). | 12 hours (minimum) | Smoking | 12.2 ± 6.5  years | Not specified | NST |
| Courtney et al., 2014^45^ | **Nicotine** | - ***Imaging:*** fMRI.  - ***Modality:*** Visual (Videos).  - ***Design:*** Block.  Participants were instructed to watch and attend to the stimuli. | ***DA***:39 | **Sex**  *DA:* 25 men,15 women  **Co-occurring disorders**  No other axis I psychiatric condition other than nicotine abuse or dependence.  **Socio-economic status**  Education  DA: 14.55 years (± 3.73)  Race   - 28 Caucasian - 7 African American - 2 Asian - 3 Latino | ***Nicotine:*** Smoking-related videos filmed in a first-person point of view (i.e. writing a letter and smoking a cigarette or standing outside of a nightclub smoking a cigarette). | ***Neutral:*** Neutral videos matched for similar content except for the absence of smoking cues. | ***Assessment:*** During fMRI.  ***Scale:*** During the urge-rating period, participants were asked to rate their current urge to smoke using Likert scale ranging from 1 [No urge] to 4 [Very high urge].  ***Results:*** Cigarette cues were found to be effective in eliciting greater self-reported craving compared to the neutral cues. | Not specified (abstinence not required prior to scanning) | Smoking | Not specified | Alcohol | NST |
| David et al., 2007^46^ | **Nicotine** | - ***Imaging:*** fMRI.  - ***Modality:*** Visual (Pictures).  - ***Design:*** Event-related.  Participants were instructed to watch and attend to indicate with a keypress the gender of the subject in each photograph. | ***DA***:8 | **Sex**  *DA:* all women  **Co-occurring disorders**  No other axis I psychiatric condition other than nicotine abuse or dependence.  **Socio-economic status**  Not reported | ***Nicotine:*** Smoking-related pictures (i.e. images of humans smoking cigarettes) taken from the International Smoking Image Series (ISIS). | ***Control:*** Neutral pictures (i.e. images of humans holding pens or glasses in their hands and mouths) of the same size as smoking-related pictures. | ***Assessment****:* Before and after stimulus presentation during fMRI.  ***Scale:*** The Shiffman-Jarvik Craving Scale (SJCS) consists of five items; subjects are asked to rate each item from 0–100 (total 500 maximum).  ***Results:*** Average SJCS scores were not significantly different before and after the scans. | Abstinence: 12 hours (minimum)  Smoking = prior to scanning | Smoking | 38 ± 7.1  years | Not specified | NST |
| De Pirro et al., 2018^12^ | **Cocaine and heroin** | - ***Imaging:*** fMRI.  - ***Modality:*** Imagery.  - ***Design****:* Block.  Participants were instructed to recall a typical drug experience and to rate the affective state produced by heroin versus cocaine in two settings (at home vs outside the home). | ***DA:*** 20 | **Sex**  *DA:* all men.  **Co-occurring disorders**  No other axis I psychiatric condition other than cocaine or heroin abuse or dependence.  **Socio-economic status**  Employment: 17 employed, 3 unemployed.  Education: 13.6 years (± 3.31). | ***Cocaine:*** Scripts of situations in which cocaine is used (at home/outside home). ***Heroin:*** Scripts of situations in which heroin is used (at home/outside home). | ***Neutral:*** Scripts of relaxing situations (at home or in their usual club). | No assessment of cue-induced craving. Only vividness of imagery during fMRI is assessed. | Not specified | Cocaine and heroin: intranasal, smoking, intravenously | Cocaine: 15.25 years  Heroin: 13.20 years | Methadone, heroin, cocaine | TS (recruited from drug rehabilitation center) |
| Duncan et al., 2007^47^ | **Cocaine** | - ***Imaging:*** fMRI.  - ***Modality:*** Imagery.  - ***Design:*** Block.  Participants were told to mentally reenact personalized scripts about cocaine use and a neutral experience both with and without a stressor present (anticipation of electrical shock). | ***DA:*** 10 | **Sex**  *DA:* all men.  **Co-occurring disorders**  No other axis I psychiatric condition other than cocaine abuse or dependence, with the exception of substance-induced mood disorder or substance-induced mood disorder with psychotic features.  **Socio-economic status**  Education: 14.2 years (± 1.5). | ***Cocaine:*** Produced from a self-reported sensations checklist and from narratives of environmental contexts of personal drug use experiences. | ***Neutral:*** Script consisting of an emotion- and drug-neutral experience (getting up and dressing in the morning). | ***Assessment:*** During fMRI Scans, at the end of each baseline period, immediately after completion of the neutral scripts, and again after completion of the cocaine scripts. ***Scale:*** 100-point visual analogue scale ranging from 1 [Not at all] to 100 [The most I’ve ever felt] assessing the craving level. ***Results:*** Craving responses were higher after cocaine scripts compared to baseline and compared to the neutral scripts. | 8 ± 4.9 days | Smoking  (freebase, crack) | 15.9 ± 6.2 years | Not specified | TS |
| Elton et al., 2015^48^ | **Cocaine** | - ***Imaging:*** fMRI.  - ***Modality:*** Imagery.  - ***Design:*** Block.  Subjects were instructed to relate to previous personal experiences associated with the personalized script. | ***DA:*** 38 | **Sex**  *DA:* all men.  **Co-occurring disorders**  No other axis I psychiatric condition other than cocaine abuse or dependence.  **Socio-economic status**  Education:   - patients with a history of childhood maltreatment: 12.7 years (± 1.7); - patients without a history of childhood maltreatment: 12.2 years (± 1.1). | ***Cocaine:*** Personalized drug use script. | ***Neutral:*** Script describing nature scenes (beach, forest, lake, river). | ***Assessment:*** After each imagery script. ***Scale:*** Participant rated their cocaine craving and the vividness of the mental image on a scale ranging from 0 to 10.  ***Results:*** The average reported urge to use cocaine did not meet the statistical significance for cocaine craving compared to neutral scripts. The cocaine script condition produced significantly greater ratings of vividness of the related mental image compared to the neutral script condition. | Urine screening detected recent use of cocaine and other drugs of abuse | Not specified | 14 years (averaged between maltreated and non-maltreated) | Cigarettes, alcohol, marijuana, | NST |
| Falcone et al., 2016^49^ | **Nicotine** | - ***Imaging:*** fMRI.  - ***Modality:*** Visual (Pictures).  - ***Design:*** Event-related.  Participants were instructed to watch and attend to the stimuli. | ***DA***  SlowMetab: 30  NormalMetab: 39 | **Sex**  *DA_ SlowMetab*: 19 men, 11 women  DA_ NormalMetab: 19 men, 20 women  **Co-occurring disorders**  No other axis I psychiatric condition other than nicotine abuse or dependence.  **Socio-economic status**  *DA SlowMetab*  Post- secondary education: 22/30  *DA NormalMetab*  Post- secondary education: 26/39  *DA SlowMetab*  Race   - 21 African American - 7 Caucasian - 2 Other   *DA NormalMetab*  Race   - 18 African American - 19 Caucasian - 1 Other - 1 Not reported | ***Nicotine:*** Smoking-related pictures (i.e. images of people smoking or images of smoking-related objects, such as cigarettes or ashtrays). | ***Neutral:*** Neutral pictures (i.e. images of people engaged in everyday tasks or unrelated objects, such as pencils) matched for visual features such as size, shape, and luminosity. | ***Assessment:*** Before, during and after fMRI.  ***Scale:*** A two-item subjective craving questionnaire was administered: participants were asked to rate the degree of craving on a scale ranging from 0 [Not at all] to 10 [Extremely].  ***Results:*** Slow metabolizers reported significantly less craving in both satiety and abstinence conditions compared to normal metabolizers, but increases in craving between the smoking session and the abstinence session were not different between slow and normal metabolizers. | Abstinence: 24 hours  Smoking = 1 hour | Smoking | Not specified | None | TS |
| Garavan et al., 2000^15^ | **Cocaine** | - ***Imaging:*** fMRI.  - ***Modality:*** Visual (Videos).  - ***Design:*** Block.  Participants were instructed to watch and attend to the stimuli. After each video, subjects performed a working memory task unrelated to cue-reactivity. | ***DA:*** 17  ***HC:*** 14 | **Sex**  *DA:* 14 men, 3 women; *HC****:*** 9 men, 5 women.  **Co-occurring disorders**  No other axis I psychiatric condition other than cocaine abuse or dependence.  **Socio-economic status**  Not reported. | ***Cocaine:*** People engaged in drug-specific dialogue while smoking "crack cocaine" and "drinking alcohol". | ***Nature:*** Scenic outdoor images. ***Sex:*** Explicit group heterosexual activity. | ***Assessment:*** Inside MRI scanner, after each movie. ***Scale:*** Questions focusing on subjects' responses to the movie. ***Results:*** DA showed significant higher craving composite scores compared to HC. | Not reported | Smoking  (freebase, crack) | 11 years (range:2-25) | Not specified | NST |
| George et al., 2001^50^ | **Alcohol** | - ***Imaging:*** fMRI.  - ***Modality:*** Visual (Pictures).  - ***Design:*** Block.  Participants were instructed to watch and attend to the stimuli. | ***DA***: 10  ***HC***: 10 | **Sex**  *DA:* 8 men, 2 women.  HC: 8 men, 2 women  **Co-occurring disorders**  No other axis I psychiatric condition other than alcohol abuse or dependence  **Socio-economic status**  Not reported. | ***Alcohol:*** Alcohol-related pictures (wine, beer, liquor). | ***Non-alcoholic beverages:*** Pictures of non-alcoholic beverages.  ***Control:*** Blurred alcohol-related pictures. | ***Assessment:*** Before and after the sip of alcohol (before fMRI), during cue-reactivity and after fMRI. ***Scale:*** Assessment of current craving using a visual analogue scale ranging from 0 to 100. ***Results:*** Alcoholic subjects had a higher self-reported of urge to drink alcohol compared to HC at all time points. Significant increase of craving levels across time points for both alcoholic subjects and social drinkers | 3.4 ± 2.2 days | Ingestion | Not specified | Not specified (negative urine drug screening) | NST |
| Goudriaan et al., 2010^51^ | **Nicotine** | - ***Imaging:*** fMRI.  ***- Modality:*** Visual (Pictures).  - ***Design:*** Event-related.  Participants were instructed to watch and attend to the stimuli. To ensure attentional focus, participants had to press a response button with their left index finger when a face was present in the picture and they had to press a response button with their right index finger when no face was present. | ***DA:***18   - *FTDN-High: 10* - *FTDN-Low:8*   ***HC***: 17 | **Sex**  *DA:* all men  *HC*: all men  **Co-occurring disorders**  No other axis I psychiatric condition other than nicotine abuse or dependence  **Socio-economic status**  *DA*  Education: 4.1 level (± 1.1)  *HC*  Education: 4.3 level (± 1.2) | ***Nicotine:*** Smoking-related pictures (i.e. several persons smoking, detailed image of a hand with a cigarette). | ***Neutral:*** Neutral pictures matched for complexity and similar content except for the absence of smoking cues (i.e. a hand with a magazine, persons talking). | ***Assessment:*** Before and after fMRI.  ***Scale:*** Smoking Urge Questionnaire [range 1-7]. ***Results:*** Craving for smoking before scanning was higher in the smoking group compared with healthy controls. No differences between smoking craving before and after the cue reactivity task in the group. | 16-18 hours | Smoking | Not specified | None | TS |
| Goudriann et al., 2013^52^ | **Cocaine** | - ***Imaging:*** fMRI.  - ***Modality:*** Visual (Pictures).  - ***Design:*** Block.  Participants were instructed to pay attention to the images and press a button when a target image (picture of an animal) is presented. | ***DA:*** 13  ***HC:*** 16 | **Sex**  *DA:* not reported; *HC:* not reported.  **Co-occurring disorders**  No other axis I psychiatric condition other than cocaine abuse or dependence.  **Socio-economic status**  Not reported. | ***Cocaine:*** Individuals preparing for cocaine use, individuals using cocaine, or cocaine user-related objects, cocaine paraphernalia, different ways of administration (snorting, crack use). | ***Neutral:*** Not specified. | ***Assessment:*** Before and after scanning. ***Scale:*** Craving Urge Questionnaire (CUQ): 8 items with a 7-point Likert scale ranging from 1 [Strongly disagree] to 7 [Strongly agree]. ***Results****:* No change in craving scores across conditions. | At least 3 weeks | Not specified | Not specified | Cigarettes | TS (in treatment) |
| Grusser et al., 2004^53^ | **Alcohol** | - ***Imaging:*** fMRI.  - ***Modality:*** Visual (Pictures).  - ***Design:*** Block.  Participants were instructed to watch and attend to the stimuli. | ***DA***:10  ***HC***:10 | **Sex**  *DA:* 5 men, 5 women  HC:5 men, 5 women  **Co-occurring disorders**  No other axis I psychiatric condition other than alcohol abuse or dependence (and axis-II disorders in HC).  **Socio-economic status**  Not reported | ***Alcohol:*** Alcohol-related pictures. | ***Neutral:*** Affectively-neutral pictures.  ***Control:*** Scrambled alcohol-related pictures. | ***Assessment:*** Before fMRI. ***Scale:*** Alcohol craving was measured using the Alcohol-Craving Questionnaire (ACQ).  ***Results:*** No significant differences were detected between alcohol-dependent individuals and healthy controls. | 9 ± 9 weeks  (range: 1-25) | Ingestion | Alcohol dependence onset: 31 years  Mean age: 36 years | None | TS |
| Hassani-Abharian et al., 2015^54^ | **Heroin** | - ***Imaging:*** fMRI.  - ***Modality:*** Visual (Pictures).  - ***Design:*** Block.  Participants were instructed to watch drug-related and neutral pictures. | ***DA:*** 25 | **Sex**  *DA:* all men.  **Co-occurring disorders**  No other axis I psychiatric condition other than cocaine abuse or dependence.  **Socio-economic status**  Education: 8.57 years (± 2.6)  Marital status:   - married: 15; - single: 5; - separated: 3; - divorced: 2. | ***Heroin:*** 24 pictures of heroin-related pictures (e.g., crystallized-heroin, paraphernalia, preparation, smoking, and co-smoking related cues). | ***Neutral:*** 24 pictures of neutral stimuli. | ***Assessment and Scale:*** During trial intervals, subjects verbally reported their subjective feeling of cue induced craving (CIC). After fMRI procedure, participants reported the intensity of their “need for drug use” and “drug use imagination” on a 0-100 visual analog scale. Afterwards, they completed positive and negative affect scale (PANAS) and desire for drug questionnaire (DDQ) with 3 components of “desire and intention to drug use”, “negative reinforcement” and “loss of control”. ***Results:*** Average verbally reported intensity of craving slightly increased during scanning. Correlation analysis among different self-reported variables exhibited no significant relationship except between the intensity of “drug use imagination” and “need for heroin use”, as well as between the intensity of “drug use imagination” and “verbal self- report of cue-induced craving”. | 4-6 hours | Not specified | 3 ± 1.9 years | Not specified | TS  (in-patient treatment) |
| He et al., 2018^55^ | **Cocaine** | - ***Imaging:*** fMRI.  - ***Modality:*** Visual (Pictures).  - ***Design:*** Block.  Participants performed a cue task in the scanner to measure their brain activity when watching two categories of stimuli: cocaine-related pictures and natural scene pictures. Participants were asked to passively view the images. | ***DA:*** 32  ***HC:*** 7 | **Sex**  *DA:* all men; *HC*: all men.  **Co-occurring disorders**  No other axis I psychiatric condition other than cocaine abuse or dependence.  **Socio-economic status**  Not reported. | ***Cocaine:*** Cocaine-related pictures (e.g., cocaine lines, cocaine crystals). | ***Neutral:*** Natural scenes (e.g., a garden, a tree). | No assessment of cue-induced craving. | USERS: < 1 years  ABS1: 1-5 years  ABS2: 6-10 years  ABS3: > 10 years | Not specified | Not specified | Not specified | NST |
| Hermann et al., 2006^56^ | **Alcohol** | - ***Imaging:*** fMRI.  ***- Modality:*** Visual (Pictures).  - ***Design:*** Block.  Participants were instructed to watch and attend to the stimuli | ***DA***: 10  ***HC***:10 | **Sex**  *DA:* all men HC: all men  **Co-occurring disorders**  No other axis I psychiatric condition other than alcohol abuse or dependence  **Socio-economic status**  Not reported | ***Alcohol:*** Alcohol-related pictures. | ***Neutral:*** Affectively-neutral pictures;  ***Control:*** Scrambled alcohol-related pictures. | ***Assessment:*** Before and after fMRI.  ***Scale:*** Alcohol wanting, the intention to consume alcohol, expected positive effects of alcohol consumption and whether alcohol could now improve negative feelings were assessed with 4 VAS [0-100 mm]. ***Results:*** No pre/post difference in any VAS. | 15 ± 5 days  (range: 8-21) | Ingestion | 19 ± 10 years | Not specified (negative urine drug screening) | TS (in-patient treatment) |
| Holla et al., 2014^57^ | **Alcohol** | - ***Imaging:*** fMRI.  - ***Modality:*** Visual (Pictures).  - ***Design:*** Block.  Participants were instructed to watch and attend to the stimuli, and to indicate by means of button presses whether they experienced craving for alcohol after seeing the picture or not. | **DA**:5 | **Sex**  *DA:* all men  **Co-occurring disorders**  No other axis I psychiatric condition other than alcohol abuse or dependence, except nicotine dependence.  **Socio-economic status**  *DA*  Education: 11.2 years | ***Alcohol***: Alcohol-related pictures in 5 scenarios (i.e. liquor stores, alcoholic beverage containers, glasses filled with alcohol, scenes of people sipping alcoholic beverages). | ***Non-alcoholic beverages:*** Neutral pictures (i.e. bar stores, bottles, glasses filled with non-alcoholic beverages, scene of people sipping non-alcoholic beverages). | ***Assessment:*** During fMRI. ***Scale:*** Button presses.  ***Results:*** Not specified. | 12-15 days | Ingestion | Alcohol dependence onset: 21.4 years  Mean age: 34.2 years | Cigarettes | TS |
| Hong et al. 2017^58^ | **Nicotine** | - ***Imaging:*** fMRI.  - ***Modality:*** Visual (Pictures).  - ***Design:*** Block.  Participants were instructed to watch and attend to the stimuli. | ***DA***:15  ***HC***:15 | **Sex**  *DA:* all men  HC: all men  **Co-occurring disorders**  No other axis I psychiatric condition other than nicotine abuse or dependence.  **Socio-economic status**  *DA*  Education: 11.9 years (± 3.1)  *HC*  Education: 12.0 years (± 3.7) | ***Nicotine:*** Smoking-related pictures downloaded from Google with the search terms: ‘positive smoking’, ‘negative smoking’. | ***Control:*** Pictures obtained as mosaic modification of tobacco-related images. | No assessment of cue-induced craving. | Not specified | Smoking | 16.8 ± 7.4  pack years | Alcohol | NST |
| Huang et al. 2018^59^ | **Alcohol** | - ***Imaging:*** fMRI.  - ***Modality:*** Visual (Pictures).  - ***Design:*** Block.  Participants were instructed to watch and attend to the stimuli. | ***DA***:11 | **Sex**  *DA:* 8 men, 3 women  **Co-occurring disorders**  No other axis I psychiatric condition other than alcohol abuse or dependence.  **Socio-economic status**  Not reported | ***Alcohol:*** Alcohol-related pictures. | ***Non-alcoholic beverages:*** Pictures of non-alcoholic beverages.  ***Control:*** Blurred pictures. | ***Assessment:*** Before fMRI. ***Scale:*** Assessment of current craving using a Numeric Rating Scale (question: “How much do you desire for alcohol?”). The range of the scale is not reported.  ***Results:*** No statistics is provided. Mean subjective alcohol craving reported in Table 1 is 8.32 (standard deviation: 1.87). | 24 hours (minimum) | Ingestion | Not specified | Not specified | NST |
| Janes et al. 2015^60^ | **Nicotine** | - ***Imaging:*** fMRI.  - ***Modality:*** Visual (Pictures).  - ***Design:*** Event-related.  Participants were instructed to watch and attend to the stimuli. To ensure attentional focus, participants were instructed to press a button when they see a target image to ensure attention to the task. | ***DA***:17 | **Sex**  *DA:* 8 men, 9 women  **Co-occurring disorders**  No other axis I psychiatric condition other than nicotine abuse or dependence.  **Socio-economic status**  Education  DA: 15.3 years (± 2.1) | ***Nicotine:*** Smoking-related pictures comprised of 3 categories: people smoking, people holding cigarettes and smoking-related items such as cigarettes. | ***Neutral:*** Neutral pictures matched for content with respect to people, body parts and manipulated objects (i.e. pens or paint brushes). | ***Assessment:*** Before the fMRI.  ***Scale:*** Brief Questionnaire for Smoking Urges (Brief-QSU). ***Results:*** Data not reported. | 1.5 hours | Smoking | 9.5 ± 5.5  pack years | None | NST |
| Janse Van Rensburg et al., 2009^61^ | **Nicotine** | - ***Imaging:*** fMRI.  ***- Modality:*** Visual (Pictures).  - ***Design:*** Crossover.  Participants were instructed to watch and attend to the stimuli. Participants were asked to press a button to ensure attentional focus. | ***DA***:10 | **Sex**  *DA:* 6 men, 4 women  **Co-occurring disorders**  No other axis I psychiatric condition other than nicotine abuse or dependence  **Socio-economic status**  Not reported | ***Nicotine:*** Smoking-related pictures (i.e. images of humans smoking cigarettes) taken from the International Smoking Image Series (ISIS). | ***Neutral:*** Neutral pictures (i.e. images of humans holding pens or glasses in their hands and mouths) of the same size as smoking-related pictures. | ***Assessment:*** Before fMRI.  ***Scale:*** Three times before fMRI session the participants verbally rated their agreement to the statement ‘I have a desire to smoke’ [1 = ‘strongly disagree’, 7 = ‘strongly agree’].  ***Results***: Baseline ‘desire to smoke’ was 4.4 (0.58) in the control condition. | 15 hours | Smoking | 8.2 ± 5.5  years | Not specified | NST |
| Kaag et al., 2018^14^ | **Cocaine** | - ***Imaging:*** fMRI.  - ***Modality:*** Visual (Pictures).  - ***Design:*** Event-related.  Participants were instructed to watch and attend to the stimuli. To ensure participants' attention, they were instructed to press a key on a response box when they see a picture of an animal. | ***DA:*** 59  ***HC:*** 58 | **Sex**  *DA:* all men; *HC****:*** all men.  **Co-occurring disorders**  No other axis I psychiatric condition other than cocaine abuse or dependence.  **Socio-economic status**  Not reported. | ***Cocaine:*** Photos of cocaine and individuals snorting cocaine. | ***Neutral:*** Photos of individuals and objects visually matched to the cocaine pictures on color, composition and type of gesture. | ***Assessment:*** Inside MRI scanner, at baseline and at the end of the experimental paradigm. ***Scale:*** Visual analogue scale ("How much do you crave for cocaine right now?") ranging from 0 [Not at all] to 10 [Extremely]. ***Results:*** Only in DA, and not in HC, craving for cocaine significantly increased during the cue-reactivity task. | 33/59 positive on drug urine screening | Intranasal | 6 ± 12 years | Cannabis, MDMA, alcohol | NST |
| Kilts et al., 2001^62^ | **Cocaine** | - ***Imaging:*** PET (O^15^).  - ***Modality:*** Imagery.  - ***Design:*** Block.  Participants are instructed to mentally rehearse the situation illustrated by the individual imagery script. | ***DA:*** 8 | **Sex**  *DA:* all men.  **Co-occurring disorders**  No other axis I and II psychiatric condition other than cocaine abuse or dependence; 1 subject fulfilled DSM-IV criteria for nicotine dependence and 1 subject fulfilled DSM-IV criteria for marijuana abuse.  **Socio-economic status**  Not reported. | ***Cocaine:*** Script-guided imagery of autobiographical memories. Participants described vivid episodic memories of ritualistic acts of cocaine use and anticipatory arousal. | ***Neutral:*** Neutral episodic memory recall, selected from either a beach or forest scene. ***Anger:*** Anger-episodic memory recall, selected from either a beach or forest scene. | ***Assessment:*** Following offset of the scanner. ***Scale:*** The inductive properties of the imagery scripts were evaluated for each condition using 0 to 10 visual analogue scales. For the cocaine scripts, subjects self-rated the vividness of the mental image (“how vivid was the image?”) and the experience of drug craving (“how strong was the urge to use?”).  ***Results:*** Anger-related script imagery was associated with mild to absent self-rated cocaine craving and it was significantly less compared to cocaine use scripts. | 3-30 days  (negative urine screening before fMRI) | Smoking  (freebase, crack) | 10 ± 6 years | Cigarettes (1/8 subjects), marijuana (1/8 subjects) | TS (inpatient treatment) |
| Kim et al., 2014^63^ | **Alcohol** | - ***Imaging:*** fMRI.  - ***Modality:*** Visual (Videos).  - ***Design:*** Block.  Participants were instructed to watch and attend to the stimuli. | ***DA***: 38  ***HC***: 26 | **Sex**  *DA:* 27 men, 11 women  HC:20 men, 6 women  **Co-occurring disorders**  No other axis I psychiatric condition other than alcohol abuse or dependence (DA group) or nicotine (both groups).  **Socio-economic status**  *DA*  Education: 11.6 years (± 2.5)  *HC*  Education: 12.9 years (± 3.5) | ***Alcohol:*** Video depicting alcohol-related scenes to induce craving. | ***Control:*** Blurred video segment. | ***Assessment***: Before fMRI. ***Scale***: Korean Alcohol Urge Questionnaire (AUQ-K). ***Results***: Alcoholic subjects had higher AUQ-K scores compared to healthy controls. | 3-21 days | Ingestion | 15.9 ± 9.5 years | Cigarettes | TS |
| Kober et al., 2016^64^ | **Cocaine** | - ***Imaging:*** fMRI.  - ***Modality:*** Visual (Videos).  - ***Design:*** Block.  Participants were instructed to view six videos depicting cocaine, gambling, and sad scenarios. | ***DA:*** 30 | **Sex**  *DA:* 18 men, 12 women.  **Co-occurring disorders**  No other axis I psychiatric condition other than cocaine abuse or dependence.  **Socio-economic status**  Education:   - men: 13.06 years (± 1.14); - women: 12.30 years (± 1.06). | ***Cocaine:*** Videos including cocaine use (e.g., presentation of cocaine and paraphernalia, preparation of a “crack hit,” repeated smoking, and getting a rush with a description of how good it was). | ***Gambling:*** Videos including gambling experiences (e.g., spinning slot machines, rolling a dice).  ***Sad:*** Videos describing sad experiences (e.g., death of a close family member, divorce). | ***Assessment:*** After each run during fMRI. *Scale:* Urge to use cocaine and to gamble rated on a scale ranging from 1 [Not at all] to 10 [A lot].  ***Results:*** Gambling videos were associated with the most intense responses compared to cocaine videos and sad videos. Cocaine videos were associated with more intense urges compared to the sad videos. | Not specified | Not specified | Not specified | Cigarettes, alcohol, marijuana | NST |
| Koopmann et al., 2018^65^ | **Alcohol** | - ***Imaging:*** fMRI.  - ***Modality:*** Visual (Pictures).  - ***Design:*** Block.  Participants were instructed to watch and attend to the stimuli. | ***DA***: 41 | **Sex**  *DA:* 30 men, 11 women  **Co-occurring disorders**  No other axis I psychiatric condition other than alcohol abuse or dependence, except from nicotine dependence in the last 12 months  **Socio-economic status**  Not reported | ***Alcohol:*** Alcohol-related pictures (wine, beer, spirit). | ***Neutral:*** Neutral objects. | ***Assessment:*** During fMRI, after each block. ***Scale:*** Assessment of current craving using a visual analogue scale ranging from 0 [No craving at all] to 100 [Very intense craving].  ***Results:*** No statistics is provided. Mean subjective alcohol cue-induced craving reported in Table 1 is 8.1 (standard deviation: 12.0). | 10.1 ± 5.6 days | Ingestion | Not specified | Cigarettes | TS  (inpatient treatment) |
| Kosten et al., 2006^4^ | **Cocaine** | - ***Imaging:*** fMRI.  - ***Modality:*** Visual (Videos).  - ***Design:*** Block.  Participants were instructed to watch the videos and rate their craving. | ***DA:*** 17 | **Sex**  *DA:* 12 men, 5 women.  **Co-occurring disorders**  No other axis I psychiatric condition other than cocaine abuse or dependence.  **Socio-economic status**  Employment:   - patients who relapsed from outpatient treatment: 5 employed, 4 unemployed; - patients who didn’t relapse from outpatient treatment: 6 employed, 2 unemployed.   Education:   - patients who relapsed from outpatient treatment: 12 years (± 1.4); - patients who didn’t relapse from outpatient treatment: 13 years (± 1.0). | ***Cocaine****:* Videos of an actor pretending to smoke cocaine and get a rush. | ***Neutral:*** Videos describing vegetable prices. | ***Assessment:*** During fMRI, immediately after each video.  ***Scale:*** Numbered scale ranging from 0 to 10 to rate craving intensity. ***Results:*** No significant correlation between brain activity and craving ratings. | 10 days | Not specified | 6 years | Cigarettes, alcohol | TS (participating in a clinical trial) |
| Krienke et al. 2014^66^ | **Alcohol** | - ***Imaging:*** fMRI.  - ***Modality:*** Visual (Videos).  - ***Design:*** Block.  Participants were instructed to watch and attend to the stimuli. | ***DA***:30 | **Sex**  *DA:* 23 men, 7 women  **Co-occurring disorders**  No other axis I psychiatric condition other than alcohol abuse or dependence.  **Socio-economic status**  Not reported | ***Alcohol:*** Alcohol-related videos tailored on the participant's preference (beer, wine or hard liquor). | ***Control***: neutral videos (i.e. two hands folding and coloring a paper boat; a person soldering). | ***Assessment:*** During fMRI.  ***Scale:*** The current craving level was assessed using a VAS ranging from 0 [No craving] to 10 [Heavy craving].  ***Results:*** There was a statistically significant increase in craving levels after the presentation of the alcohol-related compared to the control videos. | 27.4 days  (range: 4-70) | Ingestion | 171.9 months | Cigarettes | NST |
| Lee et al., 2013^11^ | **Alcohol** | - ***Imaging:*** fMRI.  - ***Modality:*** Visual (Pictures).  - ***Design:*** Event-related.  Participants were instructed to watch and attend to the stimuli. Participants reported craving in response to alcohol-related stimuli by means of a button press. | ***DA***: 17  ***HC***: 25 | **Sex**  *DA:* 12 men, 5 women  HC: 18 men, 5 women  **Co-occurring disorders**  No other axis I psychiatric condition other than alcohol abuse or dependence (except nicotine- or caffeine-related disorders)  **Socio-economic status**  *DA*  Education: 14.6 years (± 2.1)  *HC*  Education: 16.7 years (± 1.7) | ***Alcohol:*** Alcohol-related pictures (i.e. advertisements for alcoholic beverages and pictures of bottles of beer and soju). | ***Control***: Non-alcoholic beverage pictures (similar to the alcohol-related cues with respect to size, color and other physical properties). | ***Assessment:*** Before and after fMRI.  ***Scale:*** Participants were instructed: ‘For each of the following pictures, if you crave alcohol, press the left button. If you don’t crave alcohol, press the right button’.  ***Results:*** During the craving paradigm, the patient group reported higher craving in response to alcohol-related stimuli compared to the control group. | 39 ± 44 days | Ingestion | 8.4 ± 5.9 years | Not specified | TS (out-patient treatment) |
| Li et al., 2005^67^ | **Cocaine** | - ***Imaging:*** fMRI.  - ***Modality:*** Imagery.  - ***Design:*** Block.  Participants were told to mentally reenact personalized scripts about cocaine use, about neutral experiences and about acceptable stressful situations. | ***DA:*** 11 | **Sex**  *DA:* 5 men; 6 women.  **Co-occurring disorders**  No other axis I psychiatric condition other than cocaine abuse or dependence.  **Socio-economic status**  Education:   - men: 13.4 years (± 2.8); - women: 11.8 years (± 1.0). | ***Cocaine:*** Scripts about drug-use situations included meeting with a friend to use drugs together after being paid at work. | ***Stress:*** Acceptable stressful situations included a breakup with a significant other, a verbal argument with a significant other or family member, and employment-related stress (e.g., being fired or laid off from work).  ***Neutral:*** Include a beach scene, reading on a Sunday afternoon, an autumn day in the park. | ***Assessment:*** Before and after each trial during fMRI. ***Scale:*** Rate your craving for cocaine on a Likert scale ranging from 0 [None] to 10 [The highest level of craving ever experienced]. ***Results:*** Drug cue trials elicited a greater change in craving rating compared with stress trials. | 2 weeks (minimum) | Not specified | 12.6 years (averaged between males and females) | Not specified | TS (inpatient treatment) |
| Li et al., 2012^40^ | **Heroin** | - ***Imaging:*** fMRI.  - ***Modality:*** Visual (Pictures).  - ***Design:*** Event-related.  Participants were instructed to watch drug-related and neutral pictures passively. | ***DA:*** 24  ***HC:*** 20 | **Sex**  *DA:* all men; *HC*: all men.  **Co-occurring disorders**  No other axis I psychiatric condition other than cocaine abuse or dependence.  **Socio-economic status**  *DA*  Education: 10.9 years (± 3.1)  *HC*  Education: 10.1 years (± 2.3) | ***Heroin:*** Images of heroin injection, preparation, and paraphernalia | ***Neutral:*** Images of household objects or chores. | ***Assessment:*** Pre and post cue presentation.  ***Scale:*** Subjective heroin craving was evaluated with a visual analogue scale ranging from 0 [Least craving] to 10 [Strongest craving] using the question "To what extent do you feel the urge to use heroin?”. ***Results:*** Subjective craving scores after cue presentation were significantly higher than before cue presentation for the heroin- dependent patients. | 21.7 ± 16 days | Not specified | 78.6 ± 50.1 months | Cigarettes | TS (in-patient treatment) |
| Li et al., 2013a^68^ | **Heroin** | - ***Imaging:*** fMRI.  - ***Modality:*** Visual (Pictures).  - ***Design:*** Event-related.  Participants were instructed to watch and attend to the stimuli. | ***DA***  ***ST*** :19  ***LT:*** 18 | **Sex**  *DA:* all men  **Co-occurring disorders**  No other axis I psychiatric condition other than heroin abuse or dependence except nicotine dependence.  **Socio-economic status**  *ST*  Education: 9.7 years (±2.2)  *LT*  Education: 8.8 years (±2.3) | ***Heroin:*** Heroin-related pictures. | ***Neutral:*** Neutral drug-unrelated pictures. | ***Assessment:*** Before and after the cue-reactivity run. ***Scale:*** craving was assessed using a visual analogue scale ranging from 0 to 10 answering to the question "To what extent do you feel the urge to use heroin?". ***Results:*** Subjective craving scores after cue exposure were significantly higher than before cue exposure for the short-term abstinence group, but not for the long-term abstinence group. | Protracted abstinence: 193.3 days  Short abstinence: 23.6 days | Not specified | Protracted abstinence: 96.3 months  Short abstinence: 80.5 months | Methadone | TS (in-patient treatment) |
| Li et al., 2013b^69^ | **Heroin** | - ***Imaging:*** fMRI.  - ***Modality:*** Visual (Pictures).  - ***Design:*** Event-related.  Participants were instructed to watch and attend to the stimuli. | **DA** :14  **HC**: 15 | **Sex**  *DA:* all men HC: all men  **Co-occurring disorders**  No other axis I psychiatric condition other than heroin abuse or dependence.  **Socio-economic status**  *DA*  Education: 9.4 years (± 2.6)  *HC*  Education: 9.2 years (± 2.4) | ***Heroin:*** Heroin-related pictures. | ***Neutral:*** Neutral pictures. | ***Assessment:*** Before and after fMRI. ***Scale:*** Assessment of current craving using a visual analogue scale ranging from 0 to 10. ***Results:*** The heroin-dependent group had a higher level of subjective craving after performing the cue-induced craving task, compared to controls. | 17.6 ± 5.7 days | Not specified | 89.3 ± 50.5 months | None | TS (recruited from drug rehabilitation center) |
| Li et al., 2015^42^ | **Heroin** | - ***Imaging:*** fMRI.  - ***Modality:*** Visual (Pictures).  - ***Design:*** Event-related.  Participants were instructed to watch drug-related and neutral pictures. | ***DA:*** 44  ***HC:*** 20 | **Sex**  *DA:* all men; *HC:* all men.  **Co-occurring disorders**  No other axis I psychiatric condition other than cocaine abuse or dependence.  **Socio-economic status**  *DA*  Education:   - patients who relapsed: 9.5 years (± 2.3); - patients who didn’t relapse: 9.2 years (± 1.9).   *HC*  Education: 10.0 years (± 2.3). | ***Heroin:*** Pictures of heroin injection, preparation and paraphernalia. | ***Neutral:*** Pictures of household objects or chores. | ***Assessment:*** Pre- and post- scan.  ***Scale:*** Subjective heroin craving was evaluated with a visual analogue scale ("To what extent do you feel the urge to use heroin?”) ranging from 0 to 10. ***Results:*** Subjects who relapsed showed significantly higher craving ratings after cue exposure compared to subjects who did not relapse. | 12 hours (minimum) | Not specified | Relapsers: 69.2 months  Non-relapsers: 92.3 months | Cigarettes, methadone | TS (recruited from drug rehabilitation center) |
| Li et al., 2019^70^ | **Nicotine** | - ***Imaging:*** fMRI.  - ***Modality:*** Visual (Pictures).  - ***Design:*** Block.  Participants were instructed to watch and attend to the stimuli. | ***DA***: 24 | **Sex**  *DA:* 23 men, 1 woman  **Co-occurring disorders**  No other axis I psychiatric condition other than nicotine abuse or dependence  **Socio-economic status**  Not reported | ***Nicotine:*** Smoking-related pictures. | ***Control:*** Neutral pictures. | ***Assessment:*** Before and after fMRI in each state.  ***Scale:*** Tobacco Craving Questionnaire (TCQ).  ***Results:*** The TCQ scores were significantly decreased after the scan in the hypnotic state (hypnotic state was not included in our meta-analysis). | 2 hours  (minimum) | Smoking | 17.92 ± 6.83 years | Not specified | TS |
| Liberman et al., 2018^71^ | **Nicotine** | - ***Imaging:*** fMRI.  - ***Modality:*** Visual (Pictures).  - ***Design:*** Block.  Participants were instructed to watch and attend to the stimuli. To ensure participants attention, a yellow dot was randomly presented once during each stimulus block. When the dot appeared, participants were asked to press on the reaction button. | ***DA***: 5  ***HC***: 5 | **Sex**  *DA:* 1 man, 4 women  HC: 1 man, 4 women  **Co-occurring disorders**  No other axis I psychiatric condition other than nicotine abuse or dependence. Participants were excluded if they had alcohol or other drugs dependence.  **Socio-economic status**  Not reported | ***Nicotine:*** Smoking-related pictures (i.e. smoking persons, lit up cigarettes and hands holding cigarettes) taken from the International Smoking Image Series (ISIS). | ***Control***: Scrambled smoking-related pictures.. | ***Assessment:*** Before and after fMRI.  ***Scale:*** Brief Questionnaire for Smoking Urges (Brief-QSU).  ***Results:*** Smoking participants showed a significant increase of craving after fMRI. | Overnight abstinence | Smoking | Not specified | None | NST |
| Lou et al., 2012^8^ | **Heroin** | - ***Imaging:*** fMRI.  - ***Modality:*** Visual (Pictures).  - ***Design:*** Block.  Participants were instructed to watch drug-related and neutral pictures passively. | ***DA***  ***ST***:17 ***LT***:17 | **Sex**  *DA:* all men  **Co-occurring disorders**  No other axis I psychiatric condition other than heroin abuse or dependence.  **Socio-economic status**  *ST*  Education: 7.7 years (± 0.7)  LT  Education: 8.1 years (±0.4) | ***Heroin:*** Pictures of people using heroin. | ***Neutral:*** Pictures of people engaged in everyday activities. | ***Assessment:*** Before and after each block. ***Scale:*** The current craving level was assessed using a Likert scale ranging from 0 [Not at all] to 4 [Extremely].  ***Results:*** Craving significantly increased after heroin cues presentation in short- and long-term abstinence groups. Abstinence significantly increased craving ratings across cue types. | Short-term abstinence: 1.2 months  Long-term abstinence: 13.6 months | Not specified | Short-term abstinence: 7 years  Long-term abstinence: 8.2 years | Cigarettes | TS (in-patient treatment) |
| Mann et al., 2014^7^ | **Alcohol** | - ***Imaging:*** fMRI.  - ***Modality:*** Visual (Pictures).  - ***Design:*** Block.  Participants were instructed to watch and attend to the stimuli. | ***DA***: 73 | **Sex**  *DA:* 51 men, 22 women  **Co-occurring disorders**  No other axis I psychiatric condition other than alcohol abuse or dependence (apart from alcohol, nicotine and cannabis).  **Socio-economic status**  Not reported | ***Alcohol:*** Alcohol-related pictures. | ***Neutral:*** Affectively neutral pictures.  ***Control:*** Scrambled alcohol-related pictures (comparable in color distribution, contrast, and complexity). | ***Assessment:*** Before and after fMRI. ***Scale:*** Alcohol craving was assessed by means of a VAS.  ***Results:*** VAS results not reported. | Range: 14-21 days | Ingestion | Not specified | Cigarettes | TS  (clinical trial) |
| McBride et al., 2006^72^ | **Nicotine** | - ***Imaging:*** fMRI.  - ***Modality:*** Visual (Videos).  - ***Design:*** Block.  Participants were instructed to watch and attend to the stimuli. | ***DA***:19 | **Sex**  *DA:* 10 men, 9 women  **Co-occurring disorders**  No other axis I psychiatric condition other than nicotine abuse or dependence  **Socio-economic status**  Not reported | ***Nicotine:*** Smoking-related videos (i.e. people lighting cigarettes, smoking while socializing, blowing smoke rings) | ***Neutral:*** Neutral videos (i.e. people getting their hair cut). Videos were similar in degree of facial exposure, movement, and physical characteristics of the actors. | ***Assessment:*** During fMRI.  ***Scale:*** The craving questionnaire consisted of 7 items taken from a larger battery (Tiffany and Drobes, 1991); participants rated their agreement with each question on a VAS ranging from 0 [Not at all] to 10 [Extremely].  ***Results:*** Smoking videos were successful in significantly increasing subjective reports of craving. | Abstinence: 12 hours (minimum)  Smoking = smoke as usual | Smoking | Not specified | None | NST |
| Moran et al., 2017^73^ | **Nicotine** | - ***Imaging:*** fMRI.  - ***Modality:*** Visual (Pictures).  - ***Design:*** Event-related.  Participants were instructed to watch and attend to the stimuli. To ensure participants attention, an occasional target stimulus (picture of animal) was presented and subjects were required to press a button in response to the target. | ***DA***: 19 | **Sex**  *DA:* 9 men, 10 women  **Co-occurring disorders**  No other axis I psychiatric condition other than nicotine abuse or dependence.  **Socio-economic status**  *DA*  Education: 14.4 years (± 2.4) | ***Nicotine:*** Smoking-related pictures comprised of 3 categories: people smoking, people holding cigarettes and smoking-related items such as cigarettes. | ***Neutral:*** Neutral pictures (i.e. a person with pen in mouth, a hand holding a paintbrush and neutral items such as pens). | ***Assessment:*** Before and after fMRI. ***Scale:*** Questionnaire for Smoking Urges (QSU).  ***Results:*** Control smokers had significantly greater increase of craving than smokers with schizophrenia after fMRI (schizophrenic patients were not included in our meta-analysis). | 1.5 hours | Smoking | 9.5 ± 5.2 pack years | None | NST |
| Myrick et al. 2008^74^ | **Alcohol** | - ***Imaging:*** fMRI.  - ***Modality:*** Visual (Pictures).  - ***Design:*** Block.  Participants were instructed to watch and attend to the stimuli. | ***DA***:24 | **Sex**  *DA:* 18 men, 6 women  **Co-occurring disorders**  No other axis I psychiatric condition other than alcohol abuse or dependence  **Socio-economic status**  *DA*  Education: 13.25 years (± 2.07) | ***Alcohol:*** Alcohol-related pictures (i.e. wine, beer, spirits). | ***Non-alcoholic beverages:*** Pictures of non-alcoholic beverages.  ***Control:*** Pictures matching the alcohol cues in color and hue but lack any object recognition. | ***Assessment:*** During fMRI.  ***Scale:*** Alcohol craving was assessed by means of a VAS ranging from 0 [No craving at all] to 100 [Severe craving] after each block.  ***Results:*** Placebo-treated participants reported higher craving compared to both participants treated with the combination of naltrexone and ondansetron hydrochloride and social drinking controls. | Not specified | Ingestion | Not specified | Not specified | NST |
| Myrick et al., 2004^75^ | **Alcohol** | - ***Imaging:*** fMRI.  - ***Modality:*** Visual (Pictures).  - ***Design:*** Block.  Participants were instructed to watch and attend to the stimuli. | ***DA***: 10  ***HC***: 10 | **Sex**  *DA:* 8 men, 2 women  HC:8 men, 2 women  **Co-occurring disorders**  No other axis I psychiatric condition other than alcohol abuse or dependence (excluding caffeine).  **Socio-economic status**  *DA*  Education: 15.15 years (± 1.73)  *HC*  Education: 16.30 years (± 1.57)  DA  Race: 7 Caucasian  HC  Race: 10 Caucasian | ***Alcohol:*** Alcohol-related pictures. | ***Control***: Non-alcoholic beverage pictures. | ***Assessment:*** Before and during fMRI.  ***Scale:*** Obsessive Compulsive Drinking Scale (OCDS) before scan and a VAS [0-100 mm] for real-time craving after each block.  ***Results:*** At baseline, the alcoholic group reported a higher OCDS score compared to the control group, and alcoholic subjects showed higher ratings of craving during viewing of alcohol pictures compared to non-alcoholic and control pictures. | 24 hours (minimum) | Ingestion | Not specified | Not specified | NST |
| Park et al., 2007^16^ | **Alcohol** | - ***Imaging:*** fMRI.  - ***Modality:*** Visual (Pictures).  - ***Design:*** Block.  Participants were instructed to watch and attend to the stimuli, and to indicate their craving levels after each block. | ***DA***: 9  ***HC***: 9 | **Sex**  *DA:* 8 men, 1 woman  HC:7 men, 2 women  **Co-occurring disorders**  No other axis I psychiatric condition other than alcohol abuse or dependence  **Socio-economic status**  *DA*  Education: 14.00 years (± 1.25)  *HC*  Education: 12.9 years (± 3.5)  DA and HC: never married | ***Alcohol***: Alcohol-related pictures (Korean beer and mild liquor). | ***Control:*** Duplications of liquor pictures with blurred distortions. | ***Assessment:*** Before and after the sip of alcohol (before fMRI), during fMRI.  ***Scale:*** Obsessive Compulsive Drinking Scale (OCDS) before scan (pre- and post-sip), and a one-item Likert scale (range from 1 to 7 points) before and after scan. ***Results:*** Alcohol-dependent individuals had higher craving levels than controls pre- and post-sip. Alcoholic subjects showed higher levels of craving during visual cue compared with controls. | 24 hours (minimum) | Ingestion | Not specified | Cigarettes | NST |
| Potenza et al., 2012^76^ | **Cocaine** | - ***Imaging:*** fMRI.  - ***Modality:*** Imagery.  - ***Design:*** Block.  Participants were told to mentally reenact individualized drug-associated scripts and neutral relaxing scripts were generated in agreement with the clinical interview session. | ***DA:*** 30  ***HC:*** 36 | **Sex**  *DA:* 14 men,16 women; *HC:* 18 men, 18 women.  **Co-occurring disorders**  No other axis I psychiatric condition other than cocaine abuse or dependence, with the exception of alcohol or tobacco dependence.  **Socio-economic status**  Education:   - men: 12.1 years (± 1.8); - women: 12.6 years (± 1.1). | ***Cocaine:*** Involved descriptions of anticipation and consummatory phases of substance use (e.g., being at a bar and being offered cocaine, using cocaine with a drug-using buddy). | ***Stress:*** Including familial conflicts and work-related stress.  ***Neutral:*** Including resting on a beach or a fall day in the park. | ***Assessment:*** During scanning, pre- and post-trial.  ***Scale:*** Likert scale ranging from 0 to 10.  ***Results:*** Significant higher craving (and anxiety) ratings increase in cocaine scripts in cocaine addicted individuals compared to comparison subjects. | 2 weeks (minimum) | Not specified | 9.85 years (averaged between males and females) | Cigarettes, alcohol | TS (inpatient treatment) |
| Prisciandaro et al., 2013a^5^ | **Cocaine** | - ***Imaging:*** fMRI.  - ***Modality:*** Visual (Pictures).  - ***Design:*** Block.  Participants were instructed to watch and attend to the stimuli. | ***DA:*** 30 | **Sex**  *DA:*   - with cocaine positive Urine Drug Screen (UDS+): 5 men, 1 woman; - with cocaine negative Urine Drug Screen (UDS-): 20 men, 4 women.   **Co-occurring disorders**  No other axis I psychiatric condition other than cocaine abuse or dependence. Patients were excluded if they met DSM-IV criteria for non-cocaine substance dependence (except caffeine, nicotine, marijuana or alcohol).  **Socio-economic status**  Employment:   - UDS+: 1 employed, 5 unemployed; - UDS-: 2 employed, 22 unemployed.   Education:   - UDS+: 4 graduated from high school, 2 didn’t; - UDS-: 21 graduated from high school, 3 didn’t.   Marital status*:*   - UDS+: all married; - UDS-: 18 married, 6 unmarried. | ***Cocaine:*** Pictures of cocaine and related objects (e.g., crack pipe). | ***Neutral:*** Objects (e.g., furniture). ***Control:*** Images that lack object recognition processes, but matched by color and brightness. | ***Assessment:*** Inside MRI scanner, after each block.  ***Scale:*** Rate their craving, from 0 [None] to 4 [Severe], using a handpad.  ***Results:*** Participants with positive urine screening at one-week follow-up did not significantly differ from those with negative urine screening at follow-up in terms of subjective craving to cocaine minus neutral cues during the cue-reactivity task. | Participants were required to provide negative alcohol breath and urine drug screens for all drugs of abuse expect marijuana. | Not specified | Not reported | Cigarettes, marijuana, alcohol | TS (participating in a clinical trial) |
| Prisciandaro et al., 2013b^6^ | **Cocaine** | - ***Imaging:*** fMRI.  - ***Modality:*** Visual (Pictures).  - ***Design:*** Block.  Subjects were instructed to look at the pictures and to rate their craving. | ***DA:*** 25 | **Sex**  *DA:*   - patients treated with d-cycloserine (DCS): all men; - patients treated with placebo: 13 men, 2 women.   **Co-occurring disorders**  No other axis I psychiatric condition other than cocaine abuse or dependence. Patients were excluded if they met DSM-IV criteria for non-cocaine substance dependence (except caffeine, nicotine, marijuana or alcohol).  **Socio-economic status**  Education:   - patients treated with d-cycloserine (DCS): 9 graduated from high school, 1 didn’t; - patients treated with placebo: 13 graduated from high school, 2 didn’t.   Marital status:   - patients treated with d-cycloserine (DCS): 1 married, 9 unmarried; - patients treated with placebo: 4 married, 11 unmarried. | ***Cocaine:*** Fourteen of the 30 pictures contained both cocaine and cocaine paraphernalia (e.g., lighter, crack pipe, rolled paper money, razor), 12 contained cocaine only, and 4 depicted cocaine use. | ***Neutral:*** Pictures of neutral objects (e.g. furniture). | ***Assessment:*** During the task, after each block using a handpad. ***Scale:*** On a scale ranging from 0 [None] to 4 [Severe].  ***Results:*** Higher craving during cocaine versus neutral pictures. No significant change in craving pre- to post-fMRI. | 72 hours | Not specified | Cocaine dependence onset: 33.5 years  Mean age: 48.8 years | Cigarettes, marjuana | TS (participating in a clinical trial) |
| Prisciandaro et al., 2014^21^ | **Cocaine** | - ***Imaging:*** fMRI.  - ***Modality:*** Visual (Pictures).  - ***Design:*** Block.  Participants were instructed to watch and attend to the stimuli. | ***DA:*** 33 | **Sex**  *DA:* 33 men, 5 women.  **Co-occurring disorders**  No other axis I psychiatric condition other than cocaine abuse or dependence. Patients were excluded if they met DSM-IV criteria for non-cocaine substance dependence (except caffeine, nicotine, marijuana or alcohol).  **Socio-economic status**  Education: 18 attended college, 20 didn’t.  Marital status: 7 married, 31 unmarried. | ***Cocaine:*** Pictures of cocaine and related objects (e.g., crack pipe). | ***Neutral:*** Objects (e.g., furniture) ***Control:*** Images that lack object recognition processes, but matched by color and brightness. | ***Assessment:*** Inside MRI scanner during the task, after each block. ***Scale:*** Rate their craving, from 0 [None] to 4 [Severe], using a handpad.  ***Results:*** Subjective craving was significantly higher following cocaine pictures vs neutral objects blocks. | 72 hours | Not specified | TS = 18.11 ± 9.31 years  NST = 17.5 ± 5.79 years | Cigarettes | TS (outpatient treatment) and NST |
| Ray et al., 2015^77^ | **Cocaine** | - ***Imaging:*** fMRI.  - ***Modality:*** Visual (Videos).  - ***Design:*** Block.  Participants were instructed to watch and attend to the stimuli. | ***DA:*** 20  ***HC:*** 17 | **Sex**  *DA:* 15 men, 5 women; *HC****:*** 13 men, 4 women.  **Co-occurring disorders**  No other axis I psychiatric condition other than cocaine abuse or dependence.  **Socio-economic status**  Not reported. | ***Cocaine:*** Pictures of smokable cocaine, paraphernalia, and people smoking cocaine. | ***Neutral:*** Nature scenes. | ***Assessment:*** Inside MRI scanner, after the presentation of the first cocaine cue and neutral cue blocks. Craving ratings were collected also post-fMRI using the 10-item CCQ-Brief. ***Scale:*** Three-item version of the cocaine-craving questionnaire (CCQ-Brief). The items appeared one at a time on the screen, and participants had to press a button to rate their craving for cocaine on a seven-point scale from 1 [Strongly Disagree] to 7 [Strongly Agree]. ***Results:*** During the cue exposure task DA showed higher craving ratings to the cocaine cues compared to HC. Post-fMRI ratings showed that DA had a higher craving rating compared to HC. | 72 hours | Smoking  (freebase, crack) | 16 years  (range:3-34) | Cigarettes, alcohol | NST |
| Tabatabaei-Jafari et al., 2014^43^ | **Heroin** | - ***Imaging:*** fMRI.  - ***Modality:*** Visual (Pictures).  - ***Design:*** Block.  Participants were instructed to watch and attend to the stimuli. | ***DA***  MMT:20 ABT:20  ***HC:***20 | **Sex**  *DA:* all men; HC: all men  **Co-occurring disorders**  No other axis I psychiatric condition other than heroin abuse or dependence. Patients were excluded if they had a history of multidrug use except nicotine, alcohol, and benzodiazepines.  **Socio-economic status**  Not reported | ***Heroin:*** Heroin-related pictures. | ***Neutral:*** Neutral drug-unrelated pictures. | ***Assessment:*** Before and after fMRI. ***Scale:*** Desires for Drugs Questionnaire (DDQ): answer the questions on a 7-point Likert scale. ***Results:*** No significant change in craving levels after cue presentation in either group. | Abstinence: 9.2 months  Methadone: 11.61 months | Smoking | Abstinence: 11.35 years  Methadone: 11.05 years | Methadone, cigarettes | TS (recruited from drug rehabilitation center) |
| Tang et al., 2012^78^ | **Nicotine** | - ***Imaging:*** fMRI.  - ***Modality:*** Visual (Videos).  - ***Design:*** Block.  Participants were instructed to watch and attend to the stimuli. | ***DA Met-Fast***:15 ***DA Met-Slow***:16 | **Sex**  DA Met-Fast*:* 11 men, 4 women  DA Met-Slow: 12 men, 4 women  **Co-occurring disorders**  No other axis I psychiatric condition other than nicotine abuse or dependence.  **Socio-economic status**  Race  *DA Met-Fast*   - 8 Caucasian - 3 Asian - 4 Latin/Hispanic   *DA Met-Slow*   - 8 Caucasian - 6 Asian - 2 Latin/Hispanic | ***Nicotine:*** Smoking-related videos (i.e. lighting cigarettes, smoking while socializing). | ***Neutral:*** Neutral videos (except for the absence of smoking cues, i.e. getting hair cut), similar in terms of facial exposure, movement, and physical characteristics of the actors and did not include audio. | ***Assessment:*** Before and during fMRI.  ***Scale:*** The craving questionnaire consisted of 3 items taken from a larger battery (Tiffany and Drobes, 1991); participants rated their agreement with each question by means of a Likert scale ranging from 0 [Not at all] to 20 [Extremely].  ***Results:*** Craving scores were calculated by subtracting craving levels before scanning from craving after scanning. There were no significant differences between fast and slow nicotine metabolism groups, and normal and reduced genotype groups in changes in craving scores. | Smoking as usual | Smoking | 12 ± 5.2  years | None | NST |
| Tomasi et al., 2015^79^ | **Cocaine** | - ***Imaging:*** fMRI.  - ***Modality:*** Visual (Videos).  - ***Design:*** Block.  Participants were instructed to watch the screen continuously and to press a response button with their right thumb whenever they liked the features of the scene. | ***DA:*** 20 | **Sex**  *DA:* all men.  **Co-occurring disorders**  No other axis I psychiatric condition other than cocaine abuse or dependence.  **Socio-economic status**  Not reported. | ***Cocaine:*** Scenes that simulated purchase, preparation, and smoking of cocaine. | ***Neutral:*** Scenes about routine administrative/technical work.  ***Food:*** Scenes portraying scenes of serving and consuming ready to eat foods (i.e., meatballs, pasta, omelets, burger, and pancakes). | ***Assessment:*** During fMRI. ***Scale:*** The more the subjects pressed the response button during the food, cocaine, and neutral epochs the more they liked the features displayed in the respective scenes. The number of button presses was used to compute relative valences in a scale from 0 to 10. ***Results:*** The valences were lower for neutral cues than for food or cocaine cues but did not differ for food and cocaine cues. | Positive urine testing (cocaine use within 72 hours) | Smoking  (freebase, crack) and/or intravenously | 19.4 years (computed with “Enaguge Digitalizer”^80^ from the upper plot in Figure 8) | Cigarettes | NST |
| Volkow et al., 2010^81^ | **Cocaine** | - ***Imaging:*** PET (^18^FDG).  - ***Modality:*** Visual (Videos).  - ***Design:*** Block.  Participants are instructed to look at the videos and rate their craving. | ***DA:*** 24 | **Sex**  *DA:* 21 men; 3 women.  **Co-occurring disorders**  No other axis I psychiatric condition other than cocaine abuse or dependence.  **Socio-economic status**  Education: 13 years (± 2). | ***Cocaine:*** Cocaine-cue video with no instruction to inhibit craving responses, portraying scenes that simulated purchase, preparation, and smoking of cocaine. | ***Baseline:*** Eyes open, no video exposure. | ***Assessment:*** Pre and post video exposure. ***Scale:*** Visual analogue scale ranging from 1 to 10 for self-reports of “cocaine craving” and CCQ-brief. ***Results****:* Significant increase of craving from pre to post cocaine video exposure in the no-inhibition condition. | 2.5 ± 2 days | Smoking  (freebase, crack) | 17 ± 6 years | Cigarettes | NST |
| Vollstädt-Klein et al., 2010^82^ | **Alcohol** | - ***Imaging:*** fMRI.  - ***Modality:*** Visual (Pictures).  - ***Design:*** Block.  Participants were instructed to watch and attend to the stimuli. | ***DA***:21 | **Sex**  *DA:* 12 men, 9 women  **Co-occurring disorders**  No other axis I psychiatric condition other than alcohol abuse or dependence (and tobacco)  **Socio-economic status**  Not reported | ***Alcohol:*** Alcohol-related pictures (i.e. wine, beer, spirits). | ***Control:*** Neutral stimuli taken from the International Affective Picture Series (IAPS) and were matched for color distribution and complexity to the alcohol cues. | ***Assessment:*** During fMRI after each block.  ***Scale:*** Alcohol craving was assessed by means of a VAS ranging from 0 [No craving at all] to 100 [Severe craving] after each block.  ***Results:*** VAS results not reported. | 1.2 ± 0.9 days | Ingestion | Not specified | Not specified | NST |
| Vollstädt-Klein et al., 2011^83^ | **Nicotine** | - ***Imaging:*** fMRI.  ***- Modality:*** Visual (Pictures).  - ***Design:*** Block.  Participants were instructed to watch and attend to the stimuli. After the scanning session participants performed an unannounced recognition task, to test whether they paid sufficient attention to the stimuli during the fMRI session. | ***DA***:22  ***HC***:21 | **Sex**  *DA:* all men  *HC*: all men  **Co-occurring disorders**  No other axis I psychiatric condition other than nicotine abuse or dependence  **Socio-economic status**  Not reported | ***Nicotine:*** Smoking-related advertisements taken from print media. | ***Neutral:*** Neutral advertisements taken from print media (i.e. areas household, personal hygiene and clothing) and matched to tobacco advertisings regarding complexity, colors and content. | ***Assessment:*** During fMRI.  ***Scale:*** After each block, the participants were asked to rate their craving on a VAS ranging from 0 [No craving at all] to 100 [Extreme craving].  ***Results:*** Smokers reported substantially more subjective craving after presentation of tobacco advertisement (mean 􏰓= 41.3) 􏰓 than after control advertisement (mean = 27.8). | Prior to scanning | Smoking | 14.6 ± 7.2  years | None | NST |
| Wang et al., 2014^10^ | **Heroin** | - ***Imaging:*** fMRI.  - ***Modality:*** Visual (Pictures).  - ***Design:*** Event-related.  Participants were instructed to watch and attend to the stimuli. | ***DA***  A: 15  B: 15  ***HC***: 17 | **Sex**  *DA:* all men; HC: all men  **Co-occurring disorders**  No other axis I psychiatric condition other than heroin abuse or dependence, except nicotine.  **Socio-economic status**  *DA* education*:*   - A: 9.2 years (± 1.6) - B: 9.47 years (± 2.7)   *HC* education: 10.6 ± 2.4 | ***Heroin:*** Images of heroin injection, preparation, and paraphernalia. | ***Neutral:*** Images of household objects or chores. | ***Assessment:*** Before and after fMRI. ***Scale:*** Assessment of current craving using a visual analogue scale ranging from 0 to 10. ***Results:*** No significant change in craving levels after cue presentation in either group. No significant different craving levels between the groups. | Group A: 7.92 months  Group B: 29.62 months | Not specified | Group A: 48.6 months  Group B: 49.64 months | Methadone | TS (out-patient treatment) |
| Wang et al., 2015^84^ | **Heroin** | - ***Imaging:*** fMRI.  - ***Modality:*** Visual (Pictures).  - ***Design:*** Event-related.  Participants were instructed to watch and attend to the stimuli. | **DA**: 32 | **Sex**  *DA:* 17 men, 15 women.  **Co-occurring disorders**  No other axis I psychiatric condition other than heroin abuse or dependence with the exception of opioid and nicotine dependence, non-dependent cocaine abuse and depressive disorders.  **Socio-economic status**  Education: 13.2 years (±1.9)  Race:   - 28 Caucasian - 2 African American - 2 Asian | ***Heroin:*** Images of heroin injection, paraphernalia and preparation. | ***Neutral:*** Household objects or chores, graphically and contextually matched to the heroin-related stimuli. | ***Assessment:*** Pre and post cue reactivity task. ***Scale:*** Craving for heroin were assessed on a scale ranging from 0 [Not at all] to 9 [Extremely]. ***Results:*** A significant increase of craving scores was observed across treatment groups. The magnitude of cue-induced craving was significantly reduced in the treatment group after the last drug administration. | Not specified  (fMRI after detoxification) | Intravenous | Not specified | Not specified | TS (participating in a clinical trial) |
| Wei et al., 2019^13^ | **Heroin** | - ***Imaging:*** fMRI.  - ***Modality:*** Visual (Pictures).  - ***Design:*** Event-related.  Participants were instructed to watch and attend to the stimuli. | ***DA*** *MMT:18 PA:23*  ***HC****: 20* | **Sex**  *DA:* all men; HC: all men  **Co-occurring disorders**  No other axis I psychiatric condition other than heroin abuse or dependence. Patients were excluded if they met a drug abuse history other than heroin dependence.  **Socio-economic status**  *HC*  Education***:*** 11.1 years *(*± 2.3)  *MMT*  Education :10.3 years (±2.2)  *PA*  Education :10.4 years (±2.0) | ***Heroin:*** Images of heroin injection, paraphernalia and preparation. | ***Neutral:*** Household objects or chores. | ***Assessment:*** Pre and post fMRI scan. ***Scale:*** Craving was assessed using a Likert scale ranging from 0 [Least craving] to 10 [Strongest craving] answering to the question “To what extent do you feel the urge to use heroin?”.  ***Results:*** No significant increase in craving was observed in the short- nor long-abstinence groups. | Methadone: 5.6 months  Protracted abstinence: 6 months | Not specified | Methadone:88.86 months  Protracted abstinence: 99.3 months | Cigarettes | TS (recruited from drug rehabilitation center) |
| Wrase et al., 2007^85^ | **Alcohol** | - ***Imaging:*** fMRI.  - ***Modality:*** Visual (Pictures).  - ***Design:*** Event-related.  Participants were instructed to passively view the stimuli.  Participants had to confirm every viewed picture with a button press with the right thumb. | ***DA***:13  ***HC***:16 | **Sex**  *DA:* all men HC: all men  **Co-occurring disorders**  No other axis I psychiatric condition other than alcohol abuse or dependence  **Socio-economic status**  *DA*  Education: 9.06 years (± 1.69)  *HC*  Education: 11.69 years (± 1.54) | ***Alcohol:*** Alcohol-related pictures. | ***Control***: Neutral stimuli all inanimate and matched for complexity and color with the alcohol cues. | No assessment of cue-induced craving. | 11.5 ± 7.5 days  (range: 5-37) | Ingestion | 12.69 ± 7.09 years | Cigarettes | TS (in-patient treatment) |
| Xiao et al., 2006^86^ | **Heroin** | - ***Imaging:*** fMRI.  - ***Modality:*** Visual (Pictures).  - ***Design:*** Block.  Participants were instructed to watch drug-related and neutral pictures passively for future recognition. Eye-tracking to ensure attention to the stimuli. | ***DA:*** 14 | **Sex**  *DA:* all men.  **Co-occurring disorders**  No other axis I psychiatric condition other than cocaine abuse or dependence.  **Socio-economic status**  Not reported. | ***Heroin:*** Drug-related pictures. | ***Neutral:*** Neutral scenes pictures. | ***Assessment:*** Only pre-fMRI interview. ***Scale:*** Craving measured on a scale ranging from 1 to 5.  ***Results:*** The interview indicated that participants showed a moderate (3.5) level of craving. | 8.5 hours | Not specified | 7.1 years (range: 2-16) | Not specified | TS  (in-patient treatment) |
| Yalachkov, Kaiser, Naumer, 2009^87^ | **Nicotine** | - ***Imaging:*** fMRI.  ***- Modality:*** Visual (Pictures).  - ***Design:*** Block.  Participants were instructed to watch and attend to the stimuli. | ***DA***:15  ***HC***:15 | **Sex**  *DA:* 6 men, 9 women  HC: 6 men, 9 women  **Co-occurring disorders**  No other axis I psychiatric condition other than nicotine abuse or dependence.  **Socio-economic status**  Not reported | ***Nicotine:*** Smoking-related pictures (i.e. images of humans smoking cigarettes) taken from the International Smoking Image Series (ISIS). | ***Neutral:*** Neutral pictures (i.e. images of humans holding pens or glasses in their hands and mouths) of the same size as smoking-related pictures. | No assessment of cue-induced craving. | Smoking as usual | Smoking | Not specified | Not specified | NST |
| Zanchi et al., 2015^88^ | **Nicotine** | - ***Imaging:*** fMRI.  - ***Modality:*** Visual (Videos).  - ***Design:*** Block.  Participants were instructed to watch and attend to the stimuli. | ***DA***:14  ***HC***:18 | **Sex**  *DA:* 4 men, 10 women  HC: 7 men, 11 women  **Co-occurring disorders**  No other axis I psychiatric condition other than nicotine abuse or dependence.  **Socio-economic status**  Not reported | ***Nicotine:*** Smoking-related videos filmed in a first-person point of view (i.e. writing a letter and smoking a cigarette or standing outside of a nightclub smoking a cigarette). | ***Neutral:*** Neutral videos matched for similar content except for the absence of smoking cues. | ***Assessment:*** During fMRI.  ***Scale:*** After each video participants were asked to rate the degree of craving by means of a Likert scale ranging from 0 [No craving] to 7 [High craving].  ***Results****:* Smoking videos induced higher craving in smokers than in non-smokers. | 15 minutes | Smoking | 29.3 ± 6  years | Not specified | NST |
| Zeng et al., 2018^89^ | **Heroin** | - ***Imaging:*** fMRI.  - ***Modality:*** Visual (Pictures).  - ***Design:*** Block.  Participants were instructed to watch drug-related and neutral pictures (in isolation, with tool present, with body parts engaged in drug-taking actions) and to press a button to declare that they have seen the picture clearly. | ***DA:*** 37  ***HC:*** 29 | **Sex**  *DA:* 24 men, 13 women; *HC*: 19 men, 10 women.  **Co-occurring disorders**  No other axis I psychiatric condition other than cocaine abuse or dependence.  **Socio-economic status**  *DA*  Education: 9.83 years (± 1.8).  *HC*  Education: 10.41 years (± 1.16). | ***Heroin:*** Drug images containing only the drug itself, drug use tool images consisting of pictures of a syringe and other tools for using heroin and drug using action images containing people engaged in the activities of using tools to absorb or inject heroin. | ***Neutral:*** Including granular material pictures, daily life manipulable object pictures and pictures of people engaged in activities using manipulable objects. | ***Assessment:*** Pre- and post-fMRI. ***Scale:*** Visual Analogue Scale ranging from 0 to 7 to rate the current craving for heroin. ***Results:*** No significant increase in craving ratings from pre- to post-fMRI. | 42.11 ± 21.65 months | Nor specified | 21.32 ± 47.32 months | Cigarettes, alcohol | TS (recruited from drug rehabilitation center) |
| Zhang et al., 2018^3^ | **Cocaine** | - ***Imaging:*** fMRI.  - ***Modality:*** Visual (Pictures).  - ***Design:*** Block.  Participants were asked to look at the pictures and think about how they may relate to the scenes. | ***DA:*** 23 | **Sex**  *DA:* 17 men, 6 women.  **Co-occurring disorders**  No other axis I psychiatric condition other than cocaine abuse or dependence.  **Socio-economic status**  Not reported. | ***Cocaine:*** Pictures displaying people preparing and snorting/smoking cocaine. | ***Neutral:*** Scenes of people performing various acts with similar color and complexity as inspected visually. | ***Assessment:*** Inside scanner, at the end of each block.  ***Scale:*** After each block it was assessed by means of visual analogue scale ranging from 0 [No craving] to 10 [Highest craving ever].  ***Results:*** DA reported higher craving when viewing cocaine pictures as compared with viewing neutral pictures. | 12.1 ± 5.8 days | Not specified | 16 ± 9.7 years | Cigarettes | TS (inpatient treatment) |
| Zijlstra et al., 2009^90^ | **Heroin** | - ***Imaging:*** fMRI.  - ***Modality:*** Visual (Pictures).  - ***Design:*** Block.  Participants were instructed to watch and attend to the stimuli. | ***DA:*** 12 | **Sex**  *DA:* all men  **Co-occurring disorders**  No other axis I psychiatric condition other than heroin abuse or dependence.  **Socio-economic status**  Not reported | ***Heroin:*** Pictures depicting drug preparation and objects used for the preparation for a dose of heroin for inhalation. | ***Baseline:*** Black screen with a centered white crosshair. | ***Assessment:*** Desires for Drugs Questionnaire (DDQ) at baseline during the intake, and at the end of fMRI. ***Scale:*** DDQ for desire and intention to take drugs: 7 questions with response on a 7-point Likert scale. ***Results:*** No significant increase of heroin craving was observed after cue presentation. | 8.8 weeks | Intranasal | 16 ± 6.8 years | Cigarettes | TS (in-patient treatment) |

**Table S1 | Studied included in the meta-analysis.** Brief description of the experimental paradigms included and of the population involved in the included studies. The column ‘*Cue-reactivity paradigm’* contains information about the imaging modality, type of stimuli used (pictures, videos, imagery scripts), fMRI task design (block, event-related), and instructions given to the participants. The columns *‘Sample size’* and *‘Sample characteristics’* report basic information about the sample (number of participants, sex distribution, co-occurring disorders, and socio-economic status). The columns ‘*Drug cue’* and ‘*Baseline condition’* contain information about the content of the experimental and control stimuli, respectively (only [cue > baseline] contrasts were included). The column ‘*Cue-induced craving assessment’* contains a summary of the craving assessments and eventual changes in craving due to the cue-reactivity paradigm. The column ‘*Abstinence’* reports the average time (if not otherwise specified) of abstinence before the fMRI session. The column ‘*Main route of administration’* contains information about the preferred route of administration of the substance. The column ‘*History of abuse’* report the average (if not otherwise specified) time of substance use; pack years = numbers of packs of cigarettes smoked per day * number of years smoking. The column ‘*Treatment status’* reports whether participants were treatment-seeking (TS) or not-seeking treatment (NST) at the time of the study. fMRI, functional Magnetic Resonance Imaging; PET, Positron Emission Tomography; VAS, Visual Analogue Scale

**3.2. Table S2**

|  |  | **Left hemisphere** | | | **Right hemisphere** | | | |  | |  | | **Class of substances** | | | **Treatment status** | | | **Interaction** | |
| --- | --- | --- | --- | --- | --- | --- | --- | --- | --- | --- | --- | --- | --- | --- | --- | --- | --- | --- | --- | --- |
| **Cluster ID** | **Centroid label (BA)** | **X (SD)** | **Y (SD)** | **Z (SD)** | **X (SD)** | **Y (SD)** | **Z (SD)** | **# of foci** | | **# of contributing studies** | | **Legal** | | **Illegal** | **Treatment-seeking** | | **Not-seeking treatment** | **Class of substances-by-treatment status** | |  |
| 2 | Putamen |  |  |  | 23 (2.4) | 5 (1.9) | -4 (5.6) | 8 | | 8 | | .316 | | .881 | .664 | | .618 | .313 | |  |
| 6 | Superior medial frontal gyrus (10) | -4 (9) | 58 (3.7) | 24 (4.9) |  |  |  | 9 | | 3 | | .062 | | .987 | .931 | | .206 | 1 | |  |
| 7 | Amygdala (34) |  |  |  | 25 (3.8) | 1 (3.5) | -20 (2.8) | 9 | | 5 | | .891 | | .313 | .559 | | .707 | 1 | |  |
| 8 | Hippocampus (35) |  |  |  | 23 (5.4) | -10 (2.5) | -18 (6.1) | 14 | | 8 | | .928 | | .197 | .771 | | .419 | 1 | |  |
| 18 | Precuneus (23) |  |  |  | 9 (3.6) | -52 (4.9) | 35 (5.2) | 8 | | 7 | | .316 | | .881 | .967 | | .13 | .313 | |  |
| 19 | Middle temporal gyrus (37) |  |  |  | 52 (3.6) | -57 (3.4) | 1 (6.2) | 9 | | 5 | | .891 | | .313 | .293 | | .901 | 1 | |  |
| 20 | Inferior temporal gyrus (37) |  |  |  | 51 (5.2) | -66 (3.2) | -6 (4.6) | 22 | | 10 | | .861 | | .267 | .239 | | .879 | .581 | |  |
| 21 | Inferior occipital gyrus (19) | -46 (4.5) | -70 (3.8) | -8 (3.9) |  |  |  | 17 | | 4 | | .616 ^a^ | | .583 ^a^ | .552 | | .645 | .102 | |  |
| 22 | Inferior temporal gyrus (37) | -51 (3.4) | -63 (4.2) | -5 (5.8) |  |  |  | 17 | | 8 | | .995 | | .027 ^b^ | .076 | | .977 | 1 | |  |
| 27 | Caudate | -10 (3) | 5 (2.9) | 7 (4.6) |  |  |  | 12 | | 9 | | .964 | | .134 | .121 | | .968 | 1 | |  |
| 28 | Caudate | -13 (6.7) | 17 (3.8) | 2 (4) |  |  |  | 9 | | 7 | | .687 | | .582 | .794 | | .441 | .032 ^b^ | |  |
| 29 | Ventral striatum (25) |  |  |  | 12 (3.6) | -1 (3.2) | -13 (2.8) | 8 | | 5 | | .972 | | .154 | .382 | | .858 | 1 | |  |
| 30 | Nucleus accumbens |  |  |  | 8 (3.4) | 8 (5.6) | -6 (6.1) | 19 | | 10 | | .986 | | .049 ^b^ | .597 | | .59 | 1 | |  |
| 37 | Precuneus (23) | -9 (3.7) | -57 (3.6) | 33 (3.4) |  |  |  | 12 | | 8 | | .098 | | .969 | .742 | | .474 | .052 | |  |
| 38 | Posterior cingulum (23) | -4 (3) | -50 (4.9) | 26 (3.7) |  |  |  | 19 | | 15 | | .735 | | .443 | .41 | | .762 | .225 | |  |
| 42 | Inferior parietal lobule(40) |  |  |  | 37 (4.7) | -50 (5.2) | 51 (4) | 16 | | 10 | | .74 | | .454 | .627 | | .576 | .513 | |  |
| 47 | Middle occipital gyrus (19) |  |  |  | 49 (3.7) | -77 (4.9) | 4 (4.3) | 6 | | 5 | | .7 ^a^ | | .626 ^a^ | .607 | | .716 | 1 | |  |
| 50 | Posterior cingulum (23) | -4 (6.8) | -32 (4.6) | 31 (3.3) |  |  |  | 12 | | 10 | | .447 | | .764 | .742 | | .474 | 1 | |  |
| 51 | Inferior frontal gyrus, pars opercularis (44) |  |  |  | 47 (3.4) | 9 (2.1) | 25 (4.6) | 10 | | 9 | | .989 | | .076 | .461 | | .779 | 1 | |  |
| 60 | Middle cingulum (24) |  |  |  | 1 (4.4) | 3 (8.7) | 34 (4.4) | 11 | | 8 | | .947 | | .181 | .165 | | .953 | 1 | |  |
| 61 | Cuneus | 0 (4.3) | -71 (3.4) | 29 (2.4) |  |  |  | 5 | | 3 | | .252 | | .94 | .934 | | .266 | 1 | |  |
| 62 | Calcarine scissure/Precuneus (17/30) | 0 (6.3) | -59 (5.1) | 15 (4) |  |  |  | 12 | | 6 | | .995 | | .036 ^b^ | .032 ^b^ | | .996 | 1 | |  |
| 68 | Lingual gyrus (27) | -15 (5.3) | -35 (4.8) | -6 (5.3) |  |  |  | 10 | | 9 | | .515 ^a^ | | .728 ^a^ | .461 | | .779 | 1 | |  |
| 69 | Midbrain (ventral tegmental area) | -5 (2.8) | -23 (6.7) | -9 (4.7) |  |  |  | 12 | | 9 | | .995 | | .036 ^b^ | .032 ^b^ | | .996 | .438 | |  |
| 82 | Hippocampus (30) |  |  |  | 17 (6) | -24 (3.3) | -7 (6.3) | 11 | | 10 | | .36 | | .833 | .982 | | .067 | .343 | |  |
| 92 | Medial orbitofrontal gyrus (10) | -5 (5.6) | 52 (4.7) | -4 (4.9) |  |  |  | 15 | | 11 | | .47 | | .726 | .701 | | .5 | .006 ^c^ | |  |
| 95 | Caudate |  |  |  | 13 (5) | 14 (5.4) | 9 (5.8) | 21 | | 17 | | .923 | | .174 | .153 | | .934 | 1 | |  |
| 96 | Anterior cingulate cortex (32) | -5 (5.9) | 45 (4.5) | 18 (5.8) |  |  |  | 20 | | 9 | | .622 ^a^ | | .562 ^a^ | .087 | | .97 | .54 | |  |
| 97 | Hippocampus/amygdala (35/28) | -21 (4.2) | -5 (3.7) | -21 (3.3) |  |  |  | 17 | | 9 | | 1 | | .005 ^c^ | .076 | | .977 | 1 | |  |
| 101 | Nucleus accumbens | -7 (4) | 11 (4.9) | -10 (5.3) |  |  |  | 18 | | 10 | | .934 | | .165 | .293 | | .854 | .506 | |  |
| 103 | Perigenual anterior cingulate cortex (32) |  |  |  | 2 (4.5) | 40 (3.5) | 5 (7) | 16 | | 12 | | .344 | | .817 | .908 | | .204 | .045 ^b^ | |  |
| 107 | Inferior occipital gyrus (19) |  |  |  | 37 (5) | -78 (4.2) | -12 (3.3) | 10 | | 8 | | .989 | | .076 | .221 | | .932 | 1 | |  |
| 114 | Dorsal anterior cingulate cortex (24) | 0 (2.3) | 29 (4.2) | 22 (6.1) |  |  |  | 13 | | 7 | | .003 ^c^ | | 1 | .999 | | .004 ^c^ | 1 | |  |
| 116 | Thalamus |  |  |  | 1 (3.6) | -11 (3.7) | 4 (4.7) | 24 | | 10 | | .913 | | .181 | .158 | | .927 | .04 ^b^ | |  |

**Table S2 |** **Results of the cluster composition analysis for all the clusters overlapping with the ALE map.** For each cluster, the following information are reported: cluster ID, anatomical label according to the AAL (and Brodmann area, BA), centroid coordinates (standard deviation) in MNI, number of contributing foci, number of contributing studies, p-values for the binomial tests (class of substances, treatment status) and for the Fisher’s exact tests (class of substances-by-treatment status interactions). ^a^, undifferentiated clusters; ^b^, p < .05; ^c^, p < .01.

**3.3. Table S3**

| **Undifferentiated** | |  | | **Class of substances** | | |  | | **Treatment status** | | |  | | **Class of substances-by-treatment status** | | |  |
| --- | --- | --- | --- | --- | --- | --- | --- | --- | --- | --- | --- | --- | --- | --- | --- | --- | --- |
| **Word** | **r-value** | |  | | **Word** | **r-value** | |  | | **Word** | **r-value** | |  | | **Word** | **r-value** | |
| [traits](https://neurosynth.org/analyses/terms/traits) (personality) ^a^ | 0.09 | |  | | [tools](https://neurosynth.org/analyses/terms/tools) ^a^ | 0.098 | |  | | [midbrain](https://neurosynth.org/analyses/terms/midbrain) | 0.098 | |  | | [engagement](https://neurosynth.org/analyses/terms/engagement) ^a^ | 0.238 | |
| [medial prefrontal](file:///analyses/terms/medial%20prefrontal) | 0.088 | |  | | [ventral](https://neurosynth.org/analyses/terms/ventral) | 0.089 | |  | | [substantia](https://neurosynth.org/analyses/terms/substantia) | 0.09 | |  | | [cortex vmpfc](file:///analyses/terms/cortex%20vmpfc) | 0.236 | |
| [mpfc](https://neurosynth.org/analyses/terms/mpfc) | 0.081 | |  | | [motivational](https://neurosynth.org/analyses/terms/motivational) ^a^ | 0.078 | |  | | [gyrus cerebellum](file:///analyses/terms/gyrus%20cerebellum) | 0.082 | |  | | [vmpfc](https://neurosynth.org/analyses/terms/vmpfc) | 0.23 | |
| [cingulate cortex](file:///analyses/terms/cingulate%20cortex) | 0.077 | |  | | [midbrain](https://neurosynth.org/analyses/terms/midbrain) | 0.076 | |  | | [periaqueductal](https://neurosynth.org/analyses/terms/periaqueductal) | 0.079 | |  | | [ventromedial prefrontal](file:///analyses/terms/ventromedial%20prefrontal) | 0.188 | |
| [mentalizing](https://neurosynth.org/analyses/terms/mentalizing) ^a^ | 0.075 | |  | | [anticipation](https://neurosynth.org/analyses/terms/anticipation) ^a^ | 0.076 | |  | | [parietal occipital](file:///analyses/terms/parietal%20occipital) | 0.077 | |  | | [ventromedial](https://neurosynth.org/analyses/terms/ventromedial) | 0.184 | |
| [beliefs](https://neurosynth.org/analyses/terms/beliefs) ^a^ | 0.074 | |  | | [nucleus accumbens](file:///analyses/terms/nucleus%20accumbens) | 0.075 | |  | | [cortex precuneus](file:///analyses/terms/cortex%20precuneus) | 0.066 | |  | | [referential](https://neurosynth.org/analyses/terms/referential) (self) ^a^ | 0.161 | |
| [anterior cingulate](file:///analyses/terms/anterior%20cingulate) | 0.071 | |  | | [accumbens](https://neurosynth.org/analyses/terms/accumbens) | 0.072 | |  | | [dmn](https://neurosynth.org/analyses/terms/dmn) | 0.056 | |  | | [mpfc](https://neurosynth.org/analyses/terms/mpfc) | 0.151 | |
| [medial](https://neurosynth.org/analyses/terms/medial) | 0.069 | |  | | [addiction](https://neurosynth.org/analyses/terms/addiction) ^a^ | 0.072 | |  | | [aversive](https://neurosynth.org/analyses/terms/aversive) ^a^ | 0.056 | |  | | [connectivity](https://neurosynth.org/analyses/terms/connectivity) | 0.15 | |
| [anterior](https://neurosynth.org/analyses/terms/anterior) | 0.068 | |  | | [reward anticipation](file:///analyses/terms/reward%20anticipation) ^a^ | 0.071 | |  | | [reversal](https://neurosynth.org/analyses/terms/reversal) (learning) ^a^ | 0.055 | |  | | [midline](https://neurosynth.org/analyses/terms/midline) | 0.141 | |
| [cortex acc](file:///analyses/terms/cortex%20acc) | 0.066 | |  | | [nucleus](https://neurosynth.org/analyses/terms/nucleus) | 0.07 | |  | | [network dmn](file:///analyses/terms/network%20dmn) | 0.054 | |  | | [self referential](file:///analyses/terms/self%20referential) | 0.14 | |
| [cingulate](https://neurosynth.org/analyses/terms/cingulate) | 0.066 | |  | | [outcome](https://neurosynth.org/analyses/terms/outcome) ^a^ | 0.069 | |  | | [pcc](https://neurosynth.org/analyses/terms/pcc) | 0.052 | |  | | [cortex mpfc](file:///analyses/terms/cortex%20mpfc) | 0.138 | |
| [trait](https://neurosynth.org/analyses/terms/trait) | 0.064 | |  | | [hypothalamus](https://neurosynth.org/analyses/terms/hypothalamus) | 0.069 | |  | | [anticipatory](https://neurosynth.org/analyses/terms/anticipatory) ^a^ | 0.052 | |  | | [cingulate cortex](file:///analyses/terms/cingulate%20cortex) | 0.138 | |
| [acc](https://neurosynth.org/analyses/terms/acc) | 0.063 | |  | | [ventral striatum](file:///analyses/terms/ventral%20striatum) | 0.068 | |  | | [ventral tegmental](file:///analyses/terms/ventral%20tegmental) | 0.049 | |  | | [anterior cingulate](file:///analyses/terms/anterior%20cingulate) | 0.129 | |
| [craving](https://neurosynth.org/analyses/terms/craving) ^a^ | 0.059 | |  | | [substantia](https://neurosynth.org/analyses/terms/substantia) | 0.068 | |  | | [hypothalamus](https://neurosynth.org/analyses/terms/hypothalamus) | 0.049 | |  | | [medial prefrontal](file:///analyses/terms/medial%20prefrontal) | 0.129 | |
| [cortex mpfc](file:///analyses/terms/cortex%20mpfc) | 0.058 | |  | | [striatum](https://neurosynth.org/analyses/terms/striatum) | 0.066 | |  | | [tegmental](https://neurosynth.org/analyses/terms/tegmental) | 0.048 | |  | | [default mode](file:///analyses/terms/default%20mode) | 0.129 | |
| [visual stimuli](file:///analyses/terms/visual%20stimuli) ^a^ | 0.057 | |  | | [motivation](https://neurosynth.org/analyses/terms/motivation) | 0.066 | |  | | [heart](https://neurosynth.org/analyses/terms/heart) (rate) ^a^ | 0.047 | |  | | [default](https://neurosynth.org/analyses/terms/default) | 0.128 | |
| [contexts](https://neurosynth.org/analyses/terms/contexts) ^a^ | 0.055 | |  | | [mesolimbic](https://neurosynth.org/analyses/terms/mesolimbic) | 0.064 | |  | | [precuneus](https://neurosynth.org/analyses/terms/precuneus) | 0.045 | |  | | [cingulate](https://neurosynth.org/analyses/terms/cingulate) | 0.122 | |
| [autobiographical](https://neurosynth.org/analyses/terms/autobiographical) ^a^ | 0.053 | |  | | [subjective](https://neurosynth.org/analyses/terms/subjective) ^a^ | 0.061 | |  | | [intense](https://neurosynth.org/analyses/terms/intense) (emotion) ^a^ | 0.043 | |  | | [medial](https://neurosynth.org/analyses/terms/medial) | 0.118 | |
| [moral](https://neurosynth.org/analyses/terms/moral) (decision-making) ^a^ | 0.052 | |  | | [behavior](https://neurosynth.org/analyses/terms/behavior) ^a^ | 0.061 | |  | | [posterior cingulate](file:///analyses/terms/posterior%20cingulate) | 0.041 | |  | | [prefrontal cortex](file:///analyses/terms/prefrontal%20cortex) | 0.117 | |
| [prefrontal cortex](file:///analyses/terms/prefrontal%20cortex) | 0.051 | |  | | [monetary reward](file:///analyses/terms/monetary%20reward) ^a^ | 0.06 | |  | | [default mode](file:///analyses/terms/default%20mode) | 0.039 | |  | | [posterior cingulate](file:///analyses/terms/posterior%20cingulate) | 0.112 | |
| [emotion regulation](file:///analyses/terms/emotion%20regulation) ^a^ | 0.051 | |  | | [reward](https://neurosynth.org/analyses/terms/reward) | 0.059 | |  | | [brainstem](https://neurosynth.org/analyses/terms/brainstem) | 0.038 | |  | | [network dmn](file:///analyses/terms/network%20dmn) | 0.104 | |
| [stress](https://neurosynth.org/analyses/terms/stress) ^a^ | 0.05 | |  | | [complex](https://neurosynth.org/analyses/terms/complex) ^a^ | 0.058 | |  | | [episodic memory](file:///analyses/terms/episodic%20memory) ^a^ | 0.038 | |  | | [dmn](https://neurosynth.org/analyses/terms/dmn) | 0.103 | |
| [prefrontal](https://neurosynth.org/analyses/terms/prefrontal) | 0.049 | |  | | [lateral occipital](file:///analyses/terms/lateral%20occipital) | 0.057 | |  | | [dopaminergic](https://neurosynth.org/analyses/terms/dopaminergic) | 0.038 | |  | | [functional connectivity](file:///analyses/terms/functional%20connectivity) | 0.103 | |
| [cortex anterior](file:///analyses/terms/cortex%20anterior) | 0.048 | |  | | [dopaminergic](https://neurosynth.org/analyses/terms/dopaminergic) | 0.057 | |  | | [default](https://neurosynth.org/analyses/terms/default) | 0.037 | |  | | [pcc](https://neurosynth.org/analyses/terms/pcc) | 0.099 | |
| [emotional](https://neurosynth.org/analyses/terms/emotional) | 0.045 | |  | | [monetary](https://neurosynth.org/analyses/terms/monetary) | 0.055 | |  | | [nucleus](https://neurosynth.org/analyses/terms/nucleus) | 0.037 | |  | | [cortex posterior](file:///analyses/terms/cortex%20posterior) | 0.095 | |
| [oscillations](https://neurosynth.org/analyses/terms/oscillations) ^a^ | 0.045 | |  | | [gyrus cerebellum](file:///analyses/terms/gyrus%20cerebellum) | 0.054 | |  | | [episodic](https://neurosynth.org/analyses/terms/episodic) | 0.036 | |  | | [anterior](https://neurosynth.org/analyses/terms/anterior) | 0.092 | |
| [posterior cingulate](file:///analyses/terms/posterior%20cingulate) | 0.041 | |  | | [rewarding](https://neurosynth.org/analyses/terms/rewarding) | 0.054 | |  | | [autobiographical](https://neurosynth.org/analyses/terms/autobiographical) ^a^ | 0.035 | |  | | [ventral striatum](file:///analyses/terms/ventral%20striatum) | 0.091 | |
| [occipital cortex](file:///analyses/terms/occipital%20cortex) | 0.039 | |  | | [behaviors](https://neurosynth.org/analyses/terms/behaviors) | 0.052 | |  | | [ventral](https://neurosynth.org/analyses/terms/ventral) | 0.033 | |  | | [prefrontal](https://neurosynth.org/analyses/terms/prefrontal) | 0.09 | |
| [personality](https://neurosynth.org/analyses/terms/personality) | 0.039 | |  | | [periaqueductal](https://neurosynth.org/analyses/terms/periaqueductal) | 0.051 | |  | | [cognitive emotional](file:///analyses/terms/cognitive%20emotional) ^a^ | 0.032 | |  | | [acc](https://neurosynth.org/analyses/terms/acc) | 0.088 | |
| [object recognition](file:///analyses/terms/object%20recognition) ^a^ | 0.039 | |  | | [parietal occipital](file:///analyses/terms/parietal%20occipital) | 0.051 | |  | | [medial frontal](file:///analyses/terms/medial%20frontal) | 0.032 | |  | | [value](https://neurosynth.org/analyses/terms/value) ^a^ | 0.085 | |
| [placebo](https://neurosynth.org/analyses/terms/placebo) ^a^ | 0.039 | |  | | [probabilistic](https://neurosynth.org/analyses/terms/probabilistic) ^a^ | 0.051 | |  | | [amnestic](https://neurosynth.org/analyses/terms/amnestic) | 0.03 | |  | | [rostral](https://neurosynth.org/analyses/terms/rostral) | 0.083 | |
| [lateral occipital](file:///analyses/terms/lateral%20occipital) | 0.038 | |  | | [ventral tegmental](file:///analyses/terms/ventral%20tegmental) | 0.05 | |  | | [thalamus](https://neurosynth.org/analyses/terms/thalamus) | 0.03 | |  | | [medial orbitofrontal](file:///analyses/terms/medial%20orbitofrontal) | 0.082 | |
| [attended](https://neurosynth.org/analyses/terms/attended) ^a^ | 0.037 | |  | | [tegmental](https://neurosynth.org/analyses/terms/tegmental) | 0.049 | |  | | [cortex pcc](file:///analyses/terms/cortex%20pcc) | 0.029 | |  | | [rostral anterior](file:///analyses/terms/rostral%20anterior) | 0.081 | |
| [occipital temporal](file:///analyses/terms/occipital%20temporal) | 0.035 | |  | | [objects](https://neurosynth.org/analyses/terms/objects) ^a^ | 0.049 | |  | | [force](https://neurosynth.org/analyses/terms/force) ^a^ | 0.028 | |  | | [reward](https://neurosynth.org/analyses/terms/reward) ^a^ | 0.079 | |
| [dorsal medial](file:///analyses/terms/dorsal%20medial) | 0.035 | |  | | [object](https://neurosynth.org/analyses/terms/object) | 0.048 | |  | | [anticipation](https://neurosynth.org/analyses/terms/anticipation) | 0.028 | |  | | [medial pfc](file:///analyses/terms/medial%20pfc) | 0.078 | |
| [abuse](https://neurosynth.org/analyses/terms/abuse) ^a^ | 0.034 | |  | | [incentive delay](file:///analyses/terms/incentive%20delay) ^a^ | 0.048 | |  | | [junction tpj](file:///analyses/terms/junction%20tpj) | 0.028 | |  | | [cortex acc](file:///analyses/terms/cortex%20acc) | 0.076 | |
| [selective](https://neurosynth.org/analyses/terms/selective) | 0.033 | |  | | [rewards](https://neurosynth.org/analyses/terms/rewards) | 0.047 | |  | | [fear](https://neurosynth.org/analyses/terms/fear) ^a^ | 0.026 | |  | | [traits](https://neurosynth.org/analyses/terms/traits) (personality) ^a^ | 0.074 | |
| [default mode](file:///analyses/terms/default%20mode) | 0.033 | |  | | [form](https://neurosynth.org/analyses/terms/form) ^a^ | 0.047 | |  | | [reward](https://neurosynth.org/analyses/terms/reward) ^a^ | 0.026 | |  | | [rest](https://neurosynth.org/analyses/terms/rest) | 0.072 | |
| [dmn](https://neurosynth.org/analyses/terms/dmn) | 0.032 | |  | | [incentive](https://neurosynth.org/analyses/terms/incentive) | 0.044 | |  | | [cortex posterior](file:///analyses/terms/cortex%20posterior) | 0.025 | |  | | [dorsal attention](file:///analyses/terms/dorsal%20attention) | 0.07 | |
| [referential](https://neurosynth.org/analyses/terms/referential) | 0.032 | |  | | [sighted](https://neurosynth.org/analyses/terms/sighted) ^a^ | 0.043 | |  | | [heart rate](file:///analyses/terms/heart%20rate) | 0.024 | |  | | [ventral anterior](file:///analyses/terms/ventral%20anterior) | 0.067 | |
| [personality traits](file:///analyses/terms/personality%20traits) | 0.032 | |  | | [cortex precuneus](file:///analyses/terms/cortex%20precuneus) | 0.043 | |  | | [mild cognitive](file:///analyses/terms/mild%20cognitive) ^a^ | 0.023 | |  | | [personality](https://neurosynth.org/analyses/terms/personality) | 0.064 | |
| [mt](https://neurosynth.org/analyses/terms/mt) | 0.031 | |  | | [monetary incentive](file:///analyses/terms/monetary%20incentive) | 0.042 | |  | | [striatum](https://neurosynth.org/analyses/terms/striatum) | 0.023 | |  | | [choose](https://neurosynth.org/analyses/terms/choose) ^a^ | 0.063 | |
| [spectrum disorder](file:///analyses/terms/spectrum%20disorder) | 0.031 | |  | | [modality](https://neurosynth.org/analyses/terms/modality) | 0.042 | |  | | [resting state](file:///analyses/terms/resting%20state) | 0.022 | |  | | [cortex pcc](file:///analyses/terms/cortex%20pcc) | 0.062 | |
| [preferences](https://neurosynth.org/analyses/terms/preferences) | 0.031 | |  | | [visual motion](file:///analyses/terms/visual%20motion) | 0.041 | |  | | [pain](https://neurosynth.org/analyses/terms/pain) ^a^ | 0.022 | |  | | [arousal](https://neurosynth.org/analyses/terms/arousal) ^a^ | 0.062 | |
| [object](https://neurosynth.org/analyses/terms/object) | 0.03 | |  | | [self report](file:///analyses/terms/self%20report) | 0.041 | |  | | [tpj](https://neurosynth.org/analyses/terms/tpj) | 0.022 | |  | | [striatum](https://neurosynth.org/analyses/terms/striatum) | 0.061 | |
| [default](https://neurosynth.org/analyses/terms/default) | 0.029 | |  | | [lexical](https://neurosynth.org/analyses/terms/lexical) | 0.04 | |  | | [posterior](https://neurosynth.org/analyses/terms/posterior) | 0.022 | |  | | [task positive](file:///analyses/terms/task%20positive) ^a^ | 0.058 | |
| [stream](https://neurosynth.org/analyses/terms/stream) | 0.029 | |  | | [abstract](https://neurosynth.org/analyses/terms/abstract) | 0.038 | |  | | [medial](https://neurosynth.org/analyses/terms/medial) | 0.021 | |  | | [autobiographical](https://neurosynth.org/analyses/terms/autobiographical) ^a^ | 0.058 | |
| [resting state](file:///analyses/terms/resting%20state) | 0.028 | |  | | [posterior](https://neurosynth.org/analyses/terms/posterior) | 0.038 | |  | | [personal](https://neurosynth.org/analyses/terms/personal) ^a^ | 0.02 | |  | | [default network](file:///analyses/terms/default%20network) | 0.055 | |
| [familiar](https://neurosynth.org/analyses/terms/familiar) | 0.028 | |  | | [multisensory](https://neurosynth.org/analyses/terms/multisensory) | 0.038 | |  | | [sensation](https://neurosynth.org/analyses/terms/sensation) ^a^ | 0.019 | |  | | [rewards](https://neurosynth.org/analyses/terms/rewards) | 0.054 | |
| [social](https://neurosynth.org/analyses/terms/social) | 0.027 | |  | | [aversive](https://neurosynth.org/analyses/terms/aversive) | 0.037 | |  | | [motivational](https://neurosynth.org/analyses/terms/motivational) | 0.019 | |  | | [monetary](https://neurosynth.org/analyses/terms/monetary) ^a^ | 0.05 | |
| [resting](https://neurosynth.org/analyses/terms/resting) | 0.027 | |  | | [stream](https://neurosynth.org/analyses/terms/stream) | 0.037 | |  | | [autobiographical memory](file:///analyses/terms/autobiographical%20memory) | 0.019 | |  | | [moral](https://neurosynth.org/analyses/terms/moral) (decision-making) ^a^ | 0.047 | |
| [passively](https://neurosynth.org/analyses/terms/passively) | 0.027 | |  | | [reversal](https://neurosynth.org/analyses/terms/reversal) | 0.037 | |  | | [skin conductance](file:///analyses/terms/skin%20conductance) | 0.017 | |  | | [contexts](https://neurosynth.org/analyses/terms/contexts) ^a^ | 0.044 | |
| [readers](https://neurosynth.org/analyses/terms/readers) | 0.026 | |  | | [dmn](https://neurosynth.org/analyses/terms/dmn) | 0.036 | |  | | [painful](https://neurosynth.org/analyses/terms/painful) | 0.017 | |  | | [resting](https://neurosynth.org/analyses/terms/resting) | 0.043 | |
| [word pairs](file:///analyses/terms/word%20pairs) | 0.026 | |  | | [striatal](https://neurosynth.org/analyses/terms/striatal) | 0.036 | |  | | [autonomic](https://neurosynth.org/analyses/terms/autonomic) | 0.017 | |  | | [hubs](https://neurosynth.org/analyses/terms/hubs) | 0.042 | |
| [ventral visual](file:///analyses/terms/ventral%20visual) | 0.026 | |  | | [reinforcement](https://neurosynth.org/analyses/terms/reinforcement) | 0.036 | |  | | [midline](https://neurosynth.org/analyses/terms/midline) | 0.016 | |  | | [monetary incentive](file:///analyses/terms/monetary%20incentive) ^a^ | 0.042 | |
| [mesolimbic](https://neurosynth.org/analyses/terms/mesolimbic) | 0.026 | |  | | [inferior temporal](file:///analyses/terms/inferior%20temporal) | 0.035 | |  | | [rewards](https://neurosynth.org/analyses/terms/rewards) | 0.016 | |  | | [expectations](https://neurosynth.org/analyses/terms/expectations) ^a^ | 0.042 | |
| [chinese](https://neurosynth.org/analyses/terms/chinese) | 0.025 | |  | | [network dmn](file:///analyses/terms/network%20dmn) | 0.035 | |  | | [mesolimbic](https://neurosynth.org/analyses/terms/mesolimbic) | 0.015 | |  | | [resting state](file:///analyses/terms/resting%20state) | 0.041 | |
| [objects](https://neurosynth.org/analyses/terms/objects) | 0.025 | |  | | [money](https://neurosynth.org/analyses/terms/money) | 0.034 | |  | | [reward anticipation](file:///analyses/terms/reward%20anticipation) | 0.015 | |  | | [valence](https://neurosynth.org/analyses/terms/valence) ^a^ | 0.041 | |
| [encoding](https://neurosynth.org/analyses/terms/encoding) | 0.025 | |  | | [occipitotemporal](https://neurosynth.org/analyses/terms/occipitotemporal) | 0.034 | |  | | [retrosplenial cortex](file:///analyses/terms/retrosplenial%20cortex) | 0.014 | |  | | [remembering](https://neurosynth.org/analyses/terms/remembering) | 0.04 | |
| [characters](https://neurosynth.org/analyses/terms/characters) | 0.025 | |  | | [anticipatory](https://neurosynth.org/analyses/terms/anticipatory) | 0.034 | |  | | [vmpfc](https://neurosynth.org/analyses/terms/vmpfc) | 0.014 | |  | | [risky](https://neurosynth.org/analyses/terms/risky) | 0.039 | |
| [emotion](https://neurosynth.org/analyses/terms/emotion) | 0.024 | |  | | [medial lateral](file:///analyses/terms/medial%20lateral) | 0.033 | |  | | [basal ganglia](file:///analyses/terms/basal%20ganglia) | 0.014 | |  | | [solving](https://neurosynth.org/analyses/terms/solving) | 0.039 | |
| [extrastriate](https://neurosynth.org/analyses/terms/extrastriate) | 0.023 | |  | | [pcc](https://neurosynth.org/analyses/terms/pcc) | 0.033 | |  | | [ganglia](https://neurosynth.org/analyses/terms/ganglia) | 0.014 | |  | | [nucleus](https://neurosynth.org/analyses/terms/nucleus) | 0.037 | |
| [parahippocampal gyrus](file:///analyses/terms/parahippocampal%20gyrus) | 0.023 | |  | | [value](https://neurosynth.org/analyses/terms/value) | 0.032 | |  | | [video clips](file:///analyses/terms/video%20clips) | 0.014 | |  | | [striatal](https://neurosynth.org/analyses/terms/striatal) | 0.037 | |
| [color](https://neurosynth.org/analyses/terms/color) | 0.022 | |  | | [preferences](https://neurosynth.org/analyses/terms/preferences) | 0.032 | |  | | [electrical](https://neurosynth.org/analyses/terms/electrical) | 0.014 | |  | | [ventral medial](file:///analyses/terms/ventral%20medial) | 0.037 | |
| [occipito temporal](file:///analyses/terms/occipito%20temporal) | 0.022 | |  | | [vision](https://neurosynth.org/analyses/terms/vision) | 0.032 | |  | | [resting](https://neurosynth.org/analyses/terms/resting) | 0.012 | |  | | [personal](https://neurosynth.org/analyses/terms/personal) | 0.036 | |
| [visual cortex](file:///analyses/terms/visual%20cortex) | 0.022 | |  | | [risky](https://neurosynth.org/analyses/terms/risky) | 0.032 | |  | | [insular cortex](file:///analyses/terms/insular%20cortex) | 0.011 | |  | | [anticipation](https://neurosynth.org/analyses/terms/anticipation) | 0.036 | |
| [personal](https://neurosynth.org/analyses/terms/personal) | 0.021 | |  | | [risk taking](file:///analyses/terms/risk%20taking) | 0.032 | |  | | [conditioning](https://neurosynth.org/analyses/terms/conditioning) | 0.011 | |  | | [motivational](https://neurosynth.org/analyses/terms/motivational) | 0.036 | |
| [occipitotemporal](https://neurosynth.org/analyses/terms/occipitotemporal) | 0.021 | |  | | [occipito temporal](file:///analyses/terms/occipito%20temporal) | 0.031 | |  | | [subcortical](https://neurosynth.org/analyses/terms/subcortical) | 0.011 | |  | | [caudate](https://neurosynth.org/analyses/terms/caudate) | 0.036 | |
| [insula anterior](file:///analyses/terms/insula%20anterior) | 0.021 | |  | | [sexual](https://neurosynth.org/analyses/terms/sexual) | 0.03 | |  | | [mentalizing](https://neurosynth.org/analyses/terms/mentalizing) | 0.01 | |  | | [thinking](https://neurosynth.org/analyses/terms/thinking) | 0.035 | |
| [functional connectivity](file:///analyses/terms/functional%20connectivity) | 0.021 | |  | | [heart](https://neurosynth.org/analyses/terms/heart) | 0.03 | |  | | [nociceptive](https://neurosynth.org/analyses/terms/nociceptive) | 0.01 | |  | | [gains](https://neurosynth.org/analyses/terms/gains) | 0.034 | |
| [gestures](https://neurosynth.org/analyses/terms/gestures) | 0.02 | |  | | [early visual](file:///analyses/terms/early%20visual) | 0.03 | |  | | [memories](https://neurosynth.org/analyses/terms/memories) | 0.01 | |  | | [incentive](https://neurosynth.org/analyses/terms/incentive) | 0.034 | |
| [orthographic](https://neurosynth.org/analyses/terms/orthographic) | 0.02 | |  | | [prediction error](file:///analyses/terms/prediction%20error) | 0.029 | |  | | [striatal](https://neurosynth.org/analyses/terms/striatal) | 0.009 | |  | | [dmpfc](https://neurosynth.org/analyses/terms/dmpfc) | 0.034 | |
| [emotional valence](file:///analyses/terms/emotional%20valence) | 0.02 | |  | | [intense](https://neurosynth.org/analyses/terms/intense) | 0.029 | |  | | [retrosplenial](https://neurosynth.org/analyses/terms/retrosplenial) | 0.009 | |  | | [caudate nucleus](file:///analyses/terms/caudate%20nucleus) | 0.034 | |
| [medial frontal](file:///analyses/terms/medial%20frontal) | 0.02 | |  | | [familiar](https://neurosynth.org/analyses/terms/familiar) | 0.029 | |  | | [ventromedial](https://neurosynth.org/analyses/terms/ventromedial) | 0.008 | |  | | [cognitive emotional](file:///analyses/terms/cognitive%20emotional) | 0.033 | |
| [visual word](file:///analyses/terms/visual%20word) | 0.02 | |  | | [precuneus](https://neurosynth.org/analyses/terms/precuneus) | 0.028 | |  | | [neutral](https://neurosynth.org/analyses/terms/neutral) | 0.008 | |  | | [mental states](file:///analyses/terms/mental%20states) | 0.033 | |
| [learning](https://neurosynth.org/analyses/terms/learning) | 0.019 | |  | | [delay](https://neurosynth.org/analyses/terms/delay) | 0.028 | |  | | [ventral striatum](file:///analyses/terms/ventral%20striatum) | 0.008 | |  | | [retrosplenial](https://neurosynth.org/analyses/terms/retrosplenial) | 0.033 | |
| [occipital](https://neurosynth.org/analyses/terms/occipital) | 0.019 | |  | | [reinforcement learning](file:///analyses/terms/reinforcement%20learning) | 0.027 | |  | | [monetary](https://neurosynth.org/analyses/terms/monetary) | 0.008 | |  | | [orbitofrontal](https://neurosynth.org/analyses/terms/orbitofrontal) | 0.032 | |
| [category](https://neurosynth.org/analyses/terms/category) | 0.019 | |  | | [dorsolateral pfc](file:///analyses/terms/dorsolateral%20pfc) | 0.026 | |  | | [cingulate cortex](file:///analyses/terms/cingulate%20cortex) | 0.007 | |  | | [nucleus accumbens](file:///analyses/terms/nucleus%20accumbens) | 0.031 | |
| [parahippocampal](https://neurosynth.org/analyses/terms/parahippocampal) | 0.019 | |  | | [words](https://neurosynth.org/analyses/terms/words) | 0.026 | |  | | [amygdala](https://neurosynth.org/analyses/terms/amygdala) | 0.007 | |  | | [social interactions](file:///analyses/terms/social%20interactions) | 0.03 | |
| [self referential](file:///analyses/terms/self%20referential) | 0.018 | |  | | [action](https://neurosynth.org/analyses/terms/action) | 0.026 | |  | | [nuclei](https://neurosynth.org/analyses/terms/nuclei) | 0.006 | |  | | [retrosplenial cortex](file:///analyses/terms/retrosplenial%20cortex) | 0.029 | |
| [reactions](https://neurosynth.org/analyses/terms/reactions) | 0.018 | |  | | [recognition](https://neurosynth.org/analyses/terms/recognition) | 0.026 | |  | | [noxious](https://neurosynth.org/analyses/terms/noxious) | 0.006 | |  | | [thalamus](https://neurosynth.org/analyses/terms/thalamus) | 0.029 | |
| [word form](file:///analyses/terms/word%20form) | 0.018 | |  | | [occipito](https://neurosynth.org/analyses/terms/occipito) | 0.026 | |  | | [basal](https://neurosynth.org/analyses/terms/basal) | 0.006 | |  | | [dorsal striatum](file:///analyses/terms/dorsal%20striatum) | 0.028 | |
| [ratings](https://neurosynth.org/analyses/terms/ratings) | 0.018 | |  | | [perceptual](https://neurosynth.org/analyses/terms/perceptual) | 0.025 | |  | | [medial prefrontal](file:///analyses/terms/medial%20prefrontal) | 0.006 | |  | | [intense](https://neurosynth.org/analyses/terms/intense) | 0.028 | |
| [learning task](file:///analyses/terms/learning%20task) | 0.017 | |  | | [posterior cingulate](file:///analyses/terms/posterior%20cingulate) | 0.025 | |  | | [cingulate](https://neurosynth.org/analyses/terms/cingulate) | 0.006 | |  | | [incentive delay](file:///analyses/terms/incentive%20delay) | 0.027 | |
| [matching](https://neurosynth.org/analyses/terms/matching) | 0.017 | |  | | [conceptual](https://neurosynth.org/analyses/terms/conceptual) | 0.025 | |  | | [prefrontal cortex](file:///analyses/terms/prefrontal%20cortex) | 0.005 | |  | | [construction](https://neurosynth.org/analyses/terms/construction) | 0.027 | |
| [motion](https://neurosynth.org/analyses/terms/motion) | 0.017 | |  | | [word form](file:///analyses/terms/word%20form) | 0.025 | |  | | [semantic](https://neurosynth.org/analyses/terms/semantic) | 0.005 | |  | | [cortico](https://neurosynth.org/analyses/terms/cortico) | 0.027 | |
| [visual motion](file:///analyses/terms/visual%20motion) | 0.017 | |  | | [eating](https://neurosynth.org/analyses/terms/eating) | 0.025 | |  | | [vi](https://neurosynth.org/analyses/terms/vi) | 0.004 | |  | | [dopamine](https://neurosynth.org/analyses/terms/dopamine) | 0.027 | |
| [form](https://neurosynth.org/analyses/terms/form) | 0.017 | |  | | [gambling](https://neurosynth.org/analyses/terms/gambling) | 0.025 | |  | | [retrieval](https://neurosynth.org/analyses/terms/retrieval) | 0.004 | |  | | [accumbens](https://neurosynth.org/analyses/terms/accumbens) | 0.026 | |
| [lobe mtl](file:///analyses/terms/lobe%20mtl) | 0.017 | |  | | [avoidance](https://neurosynth.org/analyses/terms/avoidance) | 0.024 | |  | | [memory](https://neurosynth.org/analyses/terms/memory) | 0.003 | |  | | [memory retrieval](file:///analyses/terms/memory%20retrieval) | 0.026 | |
| [network dmn](file:///analyses/terms/network%20dmn) | 0.017 | |  | | [brainstem](https://neurosynth.org/analyses/terms/brainstem) | 0.024 | |  | | [prefrontal](https://neurosynth.org/analyses/terms/prefrontal) | 0.002 | |  | | [sexual](https://neurosynth.org/analyses/terms/sexual) | 0.025 | |
| [lobe](https://neurosynth.org/analyses/terms/lobe) | 0.017 | |  | | [unfamiliar](https://neurosynth.org/analyses/terms/unfamiliar) | 0.024 | |  | | [insula](https://neurosynth.org/analyses/terms/insula) | 0.002 | |  | | [emotion](https://neurosynth.org/analyses/terms/emotion) | 0.025 | |
| [valence](https://neurosynth.org/analyses/terms/valence) | 0.016 | |  | | [verbs](https://neurosynth.org/analyses/terms/verbs) | 0.024 | |  | | [controls](https://neurosynth.org/analyses/terms/controls) | 0.001 | |  | | [rewarding](https://neurosynth.org/analyses/terms/rewarding) | 0.025 | |
| [temporal cortex](file:///analyses/terms/temporal%20cortex) | 0.016 | |  | | [visual word](file:///analyses/terms/visual%20word) | 0.024 | |  | | [disorder](https://neurosynth.org/analyses/terms/disorder) | 0.001 | |  | | [mesolimbic](https://neurosynth.org/analyses/terms/mesolimbic) | 0.025 | |
| [cortex hippocampus](file:///analyses/terms/cortex%20hippocampus) | 0.016 | |  | | [default mode](file:///analyses/terms/default%20mode) | 0.024 | |  | | [chronic](https://neurosynth.org/analyses/terms/chronic) | 0 | |  | | [risk taking](file:///analyses/terms/risk%20taking) | 0.024 | |
| [tools](https://neurosynth.org/analyses/terms/tools) | 0.016 | |  | | [speakers](https://neurosynth.org/analyses/terms/speakers) | 0.023 | |  | | [cerebral cortex](file:///analyses/terms/cerebral%20cortex) | 0 | |  | | [posterior](https://neurosynth.org/analyses/terms/posterior) | 0.023 | |
| [mtl](https://neurosynth.org/analyses/terms/mtl) | 0.016 | |  | | [episodic memory](file:///analyses/terms/episodic%20memory) | 0.023 | |  | | [questionnaire](https://neurosynth.org/analyses/terms/questionnaire) | 0 | |  | | [regulate](https://neurosynth.org/analyses/terms/regulate) | 0.022 | |
| [visual](https://neurosynth.org/analyses/terms/visual) | 0.016 | |  | | [default](https://neurosynth.org/analyses/terms/default) | 0.022 | |  | | [reference](https://neurosynth.org/analyses/terms/reference) | 0 | |  | | [limbic](https://neurosynth.org/analyses/terms/limbic) | 0.022 | |
| [pseudowords](https://neurosynth.org/analyses/terms/pseudowords) | 0.016 | |  | | [dorsal striatum](file:///analyses/terms/dorsal%20striatum) | 0.022 | |  | | [categorization](https://neurosynth.org/analyses/terms/categorization) | 0 | |  | | [confidence](https://neurosynth.org/analyses/terms/confidence) | 0.022 | |
| [watching](https://neurosynth.org/analyses/terms/watching) | 0.015 | |  | | [languages](https://neurosynth.org/analyses/terms/languages) | 0.022 | |  | | [correction](https://neurosynth.org/analyses/terms/correction) | 0 | |  | | [losses](https://neurosynth.org/analyses/terms/losses) | 0.022 | |
| [visuo](https://neurosynth.org/analyses/terms/visuo) | 0.015 | |  | | [animal](https://neurosynth.org/analyses/terms/animal) | 0.022 | |  | | [rt](https://neurosynth.org/analyses/terms/rt) | 0 | |  | | [autobiographical memory](file:///analyses/terms/autobiographical%20memory) | 0.021 | |

**Table S3 | Full list of words identified by Neurosynth.** Full list of 100 terms identified by Neurosynth for all the CCA maps. Each term is clickable and redirects to the corresponding Neurosynth page.

^a^, words considered for discussion. In brackets are semantic complements taken from the exploration of the papers contributing to the term.

**4. Supplementary figures**

**4.1. Figure S1**


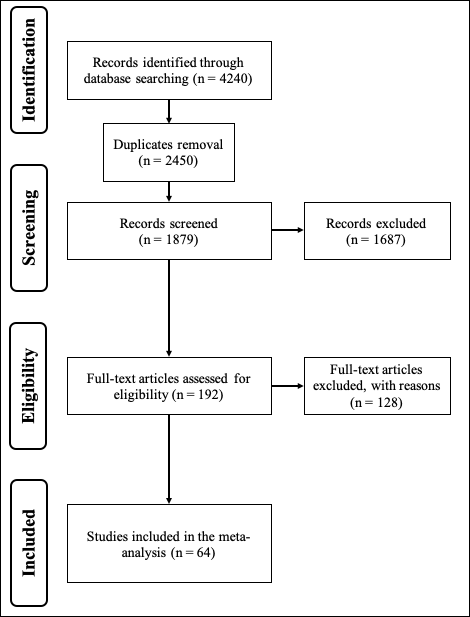


**Figure S1 | Paper selection strategy.** Flowchart of the study search and selection process that led to the identification of 64 final studies.

**4.2. Figure S2**


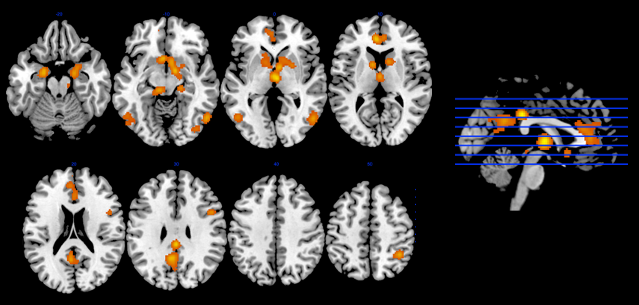


**Figure S2 | Results of the ALE meta-analysis.** The results of the ALE meta-analysis are overlaid on a structural template in MNI space. This map was used to perform the cross-validation method described in the methods section.

**4.3. Figure S3**

**
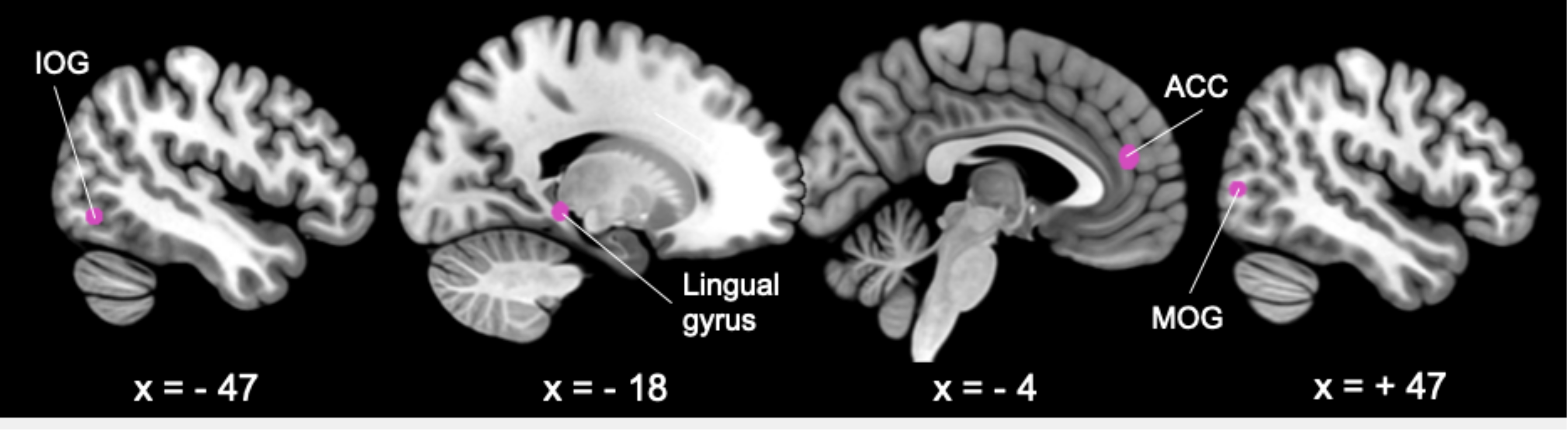
**

**Figure S3 | Clusters “undifferentiated” with respect to the factor “class of substances”.** The clusters that resulted as reliably undifferentiated across legal and illegal substances are depicted in violet. The decoder function of Neurosynth returned the following 15 terms with the highest association with the CCA map (decreasing order): traits (personality), mentalizing, beliefs, craving, visual stimuli, contexts, autobiographical, moral (decision-making), emotion regulation, oscillations, object recognition, placebo, attended, abuse. Slice coordinates are reported in MNI stereotaxic space. ACC, anterior cingulate cortex; IOG, inferior occipital gyrus; MOG, middle occipital gyrus.

**5. Supplementary references**

**5.1. References**

1 Eickhoff, S. B. *et al.* Coordinate-based activation likelihood estimation meta-analysis of neuroimaging data: a random-effects approach based on empirical estimates of spatial uncertainty. *Hum Brain Mapp* **30**, 2907-2926, doi:10.1002/hbm.20718 (2009).

2 Turkeltaub, P. E. *et al.* Minimizing within-experiment and within-group effects in Activation Likelihood Estimation meta-analyses. *Hum Brain Mapp* **33**, 1-13, doi:10.1002/hbm.21186 (2012).

3 Zhang, S., Zhornitsky, S., Angarita, G. A. & Li, C. R. Hypothalamic response to cocaine cues and cocaine addiction severity. *Addict Biol* **25**, e12682, doi:10.1111/adb.12682 (2018).

4 Kosten, T. R. *et al.* Cue-induced brain activity changes and relapse in cocaine-dependent patients. *Neuropsychopharmacology* **31**, 644-650, doi:10.1038/sj.npp.1300851 (2006).

5 Prisciandaro, J. J., Myrick, H., Henderson, S., McRae-Clark, A. L. & Brady, K. T. Prospective associations between brain activation to cocaine and no-go cues and cocaine relapse. *Drug Alcohol Depend* **131**, 44-49, doi:10.1016/j.drugalcdep.2013.04.008 (2013).

6 Prisciandaro, J. J. *et al.* Impact of DCS-facilitated cue exposure therapy on brain activation to cocaine cues in cocaine dependence. *Drug Alcohol Depend* **132**, 195-201, doi:10.1016/j.drugalcdep.2013.02.009 (2013).

7 Mann, K. *et al.* Predicting naltrexone response in alcohol-dependent patients: the contribution of functional magnetic resonance imaging. *Alcohol Clin Exp Res* **38**, 2754-2762, doi:10.1111/acer.12546 (2014).

8 Lou, M., Wang, E., Shen, Y. & Wang, J. Cue-elicited craving in heroin addicts at different abstinent time: an fMRI pilot study. *Subst Use Misuse* **47**, 631-639, doi:10.3109/10826084.2011.646381 (2012).

9 Bach, P. *et al.* Incubation of neural alcohol cue reactivity after withdrawal and its blockade by naltrexone. *Addict Biol* **25**, e12717, doi:10.1111/adb.12717 (2019).

10 Wang, Y. *et al.* Reduced responses to heroin-cue-induced craving in the dorsal striatum: effects of long-term methadone maintenance treatment. *Neurosci Lett* **581**, 120-124, doi:10.1016/j.neulet.2014.08.026 (2014).

11 Lee, E. *et al.* Neural evidence for emotional involvement in pathological alcohol craving. *Alcohol Alcohol* **48**, 288-294, doi:10.1093/alcalc/ags130 (2013).

12 De Pirro, S., Galati, G., Pizzamiglio, L. & Badiani, A. The Affective and Neural Correlates of Heroin versus Cocaine Use in Addiction Are Influenced by Environmental Setting But in Opposite Directions. *J Neurosci* **38**, 5182-5195, doi:10.1523/JNEUROSCI.0019-18.2018 (2018).

13 Wei, X. *et al.* Assessing drug cue-induced brain response in heroin dependents treated by methadone maintenance and protracted abstinence measures. *Brain Imaging Behav*, doi:10.1007/s11682-019-00051-5 (2019).

14 Kaag, A. M., Reneman, L., Homberg, J., van den Brink, W. & van Wingen, G. A. Enhanced Amygdala-Striatal Functional Connectivity during the Processing of Cocaine Cues in Male Cocaine Users with a History of Childhood Trauma. *Front Psychiatry* **9**, 70, doi:10.3389/fpsyt.2018.00070 (2018).

15 Garavan, H. *et al.* Cue-induced cocaine craving: neuroanatomical specificity for drug users and drug stimuli. *Am J Psychiatry* **157**, 1789-1798, doi:10.1176/appi.ajp.157.11.1789 (2000).

16 Park, M. S. *et al.* Brain substrates of craving to alcohol cues in subjects with alcohol use disorder. *Alcohol Alcohol* **42**, 417-422, doi:10.1093/alcalc/agl117 (2007).

17 Chase, H. W., Eickhoff, S. B., Laird, A. R. & Hogarth, L. The neural basis of drug stimulus processing and craving: an activation likelihood estimation meta-analysis. *Biol Psychiatry* **70**, 785-793, doi:10.1016/j.biopsych.2011.05.025 (2011).

18 Kühn, S. & Gallinat, J. Common biology of craving across legal and illegal drugs - a quantitative meta-analysis of cue-reactivity brain response. *Eur J Neurosci* **33**, 1318-1326, doi:10.1111/j.1460-9568.2010.07590.x (2011).

19 Engelmann, J. M. *et al.* Neural substrates of smoking cue reactivity: a meta-analysis of fMRI studies. *Neuroimage* **60**, 252-262, doi:10.1016/j.neuroimage.2011.12.024 (2012).

20 McClernon, F. J., Kozink, R. V., Lutz, A. M. & Rose, J. E. 24-h smoking abstinence potentiates fMRI-BOLD activation to smoking cues in cerebral cortex and dorsal striatum. *Psychopharmacology (Berl)* **204**, 25-35, doi:10.1007/s00213-008-1436-9 (2009).

21 Prisciandaro, J. J., McRae-Clark, A. L., Myrick, H., Henderson, S. & Brady, K. T. Brain activation to cocaine cues and motivation/treatment status. *Addict Biol* **19**, 240-249, doi:10.1111/j.1369-1600.2012.00446.x (2014).

22 Hanlon, C. A., Dowdle, L. T., Naselaris, T., Canterberry, M. & Cortese, B. M. Visual cortex activation to drug cues: a meta-analysis of functional neuroimaging papers in addiction and substance abuse literature. *Drug Alcohol Depend* **143**, 206-212, doi:10.1016/j.drugalcdep.2014.07.028 (2014).

23 Volkow, N. D. & Wise, R. A. How can drug addiction help us understand obesity? *Nat Neurosci* **8**, 555-560, doi:10.1038/nn1452 (2005).

24 Nutt, D., King, L. A., Saulsbury, W. & Blakemore, C. Development of a rational scale to assess the harm of drugs of potential misuse. *Lancet* **369**, 1047-1053, doi:10.1016/S0140-6736(07)60464-4 (2007).

25 Voon, V. *et al.* Neural correlates of sexual cue reactivity in individuals with and without compulsive sexual behaviours. *PLoS One* **9**, e102419, doi:10.1371/journal.pone.0102419 (2014).

26 Montorsi, F. *et al.* Apomorphine-induced brain modulation during sexual stimulation: a new look at central phenomena related to erectile dysfunction. *Int J Impot Res* **15**, 203-209, doi:10.1038/sj.ijir.3900999 (2003).

27 Kennerley, S. W., Walton, M. E., Behrens, T. E., Buckley, M. J. & Rushworth, M. F. Optimal decision making and the anterior cingulate cortex. *Nat Neurosci* **9**, 940-947, doi:10.1038/nn1724 (2006).

28 Schacht, J. P., Anton, R. F. & Myrick, H. Functional neuroimaging studies of alcohol cue reactivity: a quantitative meta-analysis and systematic review. *Addict Biol* **18**, 121-133, doi:10.1111/j.1369-1600.2012.00464.x (2013).

29 McFarland, K. & Kalivas, P. W. The circuitry mediating cocaine-induced reinstatement of drug-seeking behavior. *J Neurosci* **21**, 8655-8663 (2001).

30 McLaughlin, J. & See, R. E. Selective inactivation of the dorsomedial prefrontal cortex and the basolateral amygdala attenuates conditioned-cued reinstatement of extinguished cocaine-seeking behavior in rats. *Psychopharmacology (Berl)* **168**, 57-65, doi:10.1007/s00213-002-1196-x (2003).

31 Kalivas, P. W. & Volkow, N. D. The neural basis of addiction: a pathology of motivation and choice. *Am J Psychiatry* **162**, 1403-1413, doi:10.1176/appi.ajp.162.8.1403 (2005).

32 Wilson, S. J., Sayette, M. A. & Fiez, J. A. Prefrontal responses to drug cues: a neurocognitive analysis. *Nat Neurosci* **7**, 211-214, doi:10.1038/nn1200 (2004).

33 Jasinska, A. J., Stein, E. A., Kaiser, J., Naumer, M. J. & Yalachkov, Y. Factors modulating neural reactivity to drug cues in addiction: a survey of human neuroimaging studies. *Neurosci Biobehav Rev* **38**, 1-16, doi:10.1016/j.neubiorev.2013.10.013 (2014).

34 Berlingeri, M. *et al.* Clustering the Brain With "CluB": A New Toolbox for Quantitative Meta-Analysis of Neuroimaging Data. *Front Neurosci* **13**, 1037, doi:10.3389/fnins.2019.01037 (2019).

35 Devoto, F. *et al.* Hungry Brains: A Meta-Analytical Review of Brain Activation Imaging Studies On Food Perception and Appetite in Obese Individuals. *Neurosci Biobehav Rev*, doi:10.1016/j.neubiorev.2018.07.017 (2018).

36 Zapparoli, L., Seghezzi, S. & Paulesu, E. The What, the When, and the Whether of Intentional Action in the Brain: A Meta-Analytical Review. *Front Hum Neurosci* **11**, 238, doi:10.3389/fnhum.2017.00238 (2017).

37 Paulesu, E., Danelli, L. & Berlingeri, M. Reading the dyslexic brain: multiple dysfunctional routes revealed by a new meta-analysis of PET and fMRI activation studies. *Front Hum Neurosci* **8**, 830, doi:10.3389/fnhum.2014.00830 (2014).

38 Cattinelli, I., Borghese, N. A., Gallucci, M. & Paulesu, E. Reading the reading brain: A new meta-analysis of functional imaging data on reading. *Journal of Neurolinguistics* **26**, 214-238, doi:<https://doi.org/10.1016/j.jneuroling.2012.08.001> (2013).

39 Crepaldi, D. *et al.* Clustering the lexicon in the brain: a meta-analysis of the neurofunctional evidence on noun and verb processing. *Front Hum Neurosci* **7**, 303, doi:10.3389/fnhum.2013.00303 (2013).

40 Li, Q. *et al.* Craving correlates with mesolimbic responses to heroin-related cues in short-term abstinence from heroin: an event-related fMRI study. *Brain Res* **1469**, 63-72, doi:10.1016/j.brainres.2012.06.024 (2012).

41 McClernon, F. J., Kozink, R. V. & Rose, J. E. Individual differences in nicotine dependence, withdrawal symptoms, and sex predict transient fMRI-BOLD responses to smoking cues. *Neuropsychopharmacology* **33**, 2148-2157, doi:10.1038/sj.npp.1301618 (2008).

42 Li, Q. *et al.* Predicting subsequent relapse by drug-related cue-induced brain activation in heroin addiction: an event-related functional magnetic resonance imaging study. *Addict Biol* **20**, 968-978, doi:10.1111/adb.12182 (2015).

43 Tabatabaei-Jafari, H. *et al.* Patterns of brain activation during craving in heroin dependents successfully treated by methadone maintenance and abstinence-based treatments. *J Addict Med* **8**, 123-129, doi:10.1097/ADM.0000000000000022 (2014).

44 Cortese, B. M. *et al.* The fMRI BOLD response to unisensory and multisensory smoking cues in nicotine-dependent adults. *Psychiatry Res* **234**, 321-327, doi:10.1016/j.pscychresns.2015.10.008 (2015).

45 Courtney, K. E., Ghahremani, D. G., London, E. D. & Ray, L. A. The association between cue-reactivity in the precuneus and level of dependence on nicotine and alcohol. *Drug Alcohol Depend* **141**, 21-26, doi:10.1016/j.drugalcdep.2014.04.026 (2014).

46 David, S. P. *et al.* Effects of Acute Nicotine Abstinence on Cue-elicited Ventral Striatum/Nucleus Accumbens Activation in Female Cigarette Smokers: A Functional Magnetic Resonance Imaging Study. *Brain Imaging Behav* **1**, 43-57, doi:10.1007/s11682-007-9004-1 (2007).

47 Duncan, E. *et al.* An fMRI study of the interaction of stress and cocaine cues on cocaine craving in cocaine-dependent men. *Am J Addict* **16**, 174-182, doi:10.1080/10550490701375285 (2007).

48 Elton, A., Smitherman, S., Young, J. & Kilts, C. D. Effects of childhood maltreatment on the neural correlates of stress- and drug cue-induced cocaine craving. *Addict Biol* **20**, 820-831, doi:10.1111/adb.12162 (2015).

49 Falcone, M. *et al.* Brain Responses to Smoking Cues Differ Based on Nicotine Metabolism Rate. *Biol Psychiatry* **80**, 190-197, doi:10.1016/j.biopsych.2015.11.015 (2016).

50 George, M. S. *et al.* Activation of prefrontal cortex and anterior thalamus in alcoholic subjects on exposure to alcohol-specific cues. *Arch Gen Psychiatry* **58**, 345-352, doi:10.1001/archpsyc.58.4.345 (2001).

51 Goudriaan, A. E., de Ruiter, M. B., van den Brink, W., Oosterlaan, J. & Veltman, D. J. Brain activation patterns associated with cue reactivity and craving in abstinent problem gamblers, heavy smokers and healthy controls: an fMRI study. *Addict Biol* **15**, 491-503, doi:10.1111/j.1369-1600.2010.00242.x (2010).

52 Goudriaan, A. E., Veltman, D. J., van den Brink, W., Dom, G. & Schmaal, L. Neurophysiological effects of modafinil on cue-exposure in cocaine dependence: a randomized placebo-controlled cross-over study using pharmacological fMRI. *Addict Behav* **38**, 1509-1517, doi:10.1016/j.addbeh.2012.04.006 (2013).

53 Grüsser, S. M. *et al.* Cue-induced activation of the striatum and medial prefrontal cortex is associated with subsequent relapse in abstinent alcoholics. *Psychopharmacology (Berl)* **175**, 296-302, doi:10.1007/s00213-004-1828-4 (2004).

54 Hassani-Abharian, P. *et al.* Exploring Neural Correlates of Different Dimensions in Drug Craving Self-Reports among Heroin Dependents. *Basic Clin Neurosci* **6**, 271-284 (2015).

55 He, Q. *et al.* Presumed structural and functional neural recovery after long-term abstinence from cocaine in male military veterans. *Prog Neuropsychopharmacol Biol Psychiatry* **84**, 18-29, doi:10.1016/j.pnpbp.2018.01.024 (2018).

56 Hermann, D. *et al.* Blockade of cue-induced brain activation of abstinent alcoholics by a single administration of amisulpride as measured with fMRI. *Alcohol Clin Exp Res* **30**, 1349-1354, doi:10.1111/j.1530-0277.2006.00174.x (2006).

57 Holla, B. *et al.* Visual Image-Induced Craving for Ethanol (VICE): Development, Validation, and a Pilot fMRI Study. *Indian J Psychol Med* **36**, 164-169, doi:10.4103/0253-7176.130984 (2014).

58 Hong, J. S. *et al.* Cognitive avoidance and aversive cues related to tobacco in male smokers. *Addict Behav* **73**, 158-164, doi:10.1016/j.addbeh.2017.05.003 (2017).

59 Huang, Y., Mohan, A., De Ridder, D., Sunaert, S. & Vanneste, S. The neural correlates of the unified percept of alcohol-related craving: a fMRI and EEG study. *Sci Rep* **8**, 923, doi:10.1038/s41598-017-18471-y (2018).

60 Janes, A. C., Farmer, S., Peechatka, A. L., Frederick, B. e. B. & Lukas, S. E. Insula-Dorsal Anterior Cingulate Cortex Coupling is Associated with Enhanced Brain Reactivity to Smoking Cues. *Neuropsychopharmacology* **40**, 1561-1568, doi:10.1038/npp.2015.9 (2015).

61 Janse Van Rensburg, K., Taylor, A., Hodgson, T. & Benattayallah, A. Acute exercise modulates cigarette cravings and brain activation in response to smoking-related images: an fMRI study. *Psychopharmacology (Berl)* **203**, 589-598, doi:10.1007/s00213-008-1405-3 (2009).

62 Kilts, C. D. *et al.* Neural activity related to drug craving in cocaine addiction. *Arch Gen Psychiatry* **58**, 334-341, doi:10.1001/archpsyc.58.4.334 (2001).

63 Kim, S. M., Han, D. H., Min, K. J., Kim, B. N. & Cheong, J. H. Brain activation in response to craving- and aversion-inducing cues related to alcohol in patients with alcohol dependence. *Drug Alcohol Depend* **141**, 124-131, doi:10.1016/j.drugalcdep.2014.05.017 (2014).

64 Kober, H. *et al.* Brain Activity During Cocaine Craving and Gambling Urges: An fMRI Study. *Neuropsychopharmacology* **41**, 628-637, doi:10.1038/npp.2015.193 (2016).

65 Koopmann, A. *et al.* Ghrelin modulates mesolimbic reactivity to alcohol cues in alcohol-addicted subjects: a functional imaging study. *Addict Biol* **24**, 1066-1076, doi:10.1111/adb.12651 (2019).

66 Krienke, U. J. *et al.* Impact of alcohol-related video sequences on functional MRI in abstinent alcoholics. *Eur Addict Res* **20**, 33-40, doi:10.1159/000349909 (2014).

67 Li, C. S., Kemp, K., Milivojevic, V. & Sinha, R. Neuroimaging study of sex differences in the neuropathology of cocaine abuse. *Gend Med* **2**, 174-182, doi:10.1016/s1550-8579(05)80046-4 (2005).

68 Li, Q. *et al.* Assessing cue-induced brain response as a function of abstinence duration in heroin-dependent individuals: an event-related fMRI study. *PLoS One* **8**, e62911, doi:10.1371/journal.pone.0062911 (2013).

69 Li, Q. *et al.* Abnormal function of the posterior cingulate cortex in heroin addicted users during resting-state and drug-cue stimulation task. *Chin Med J (Engl)* **126**, 734-739 (2013).

70 Li, X. *et al.* The top-down regulation from the prefrontal cortex to insula via hypnotic aversion suggestions reduces smoking craving. *Hum Brain Mapp* **40**, 1718-1728, doi:10.1002/hbm.24483 (2019).

71 Liberman, K. *et al.* The effect of nicotine patches on craving in the brain: A functional MRI study on heavy smokers. *Medicine (Baltimore)* **97**, e12415, doi:10.1097/MD.0000000000012415 (2018).

72 McBride, D., Barrett, S. P., Kelly, J. T., Aw, A. & Dagher, A. Effects of expectancy and abstinence on the neural response to smoking cues in cigarette smokers: an fMRI study. *Neuropsychopharmacology* **31**, 2728-2738, doi:10.1038/sj.npp.1301075 (2006).

73 Moran, L. V., Betts, J. M., Ongur, D. & Janes, A. C. Neural Responses to Smoking Cues in Schizophrenia. *Schizophr Bull* **44**, 525-534, doi:10.1093/schbul/sbx085 (2018).

74 Myrick, H. *et al.* Effect of naltrexone and ondansetron on alcohol cue-induced activation of the ventral striatum in alcohol-dependent people. *Arch Gen Psychiatry* **65**, 466-475, doi:10.1001/archpsyc.65.4.466 (2008).

75 Myrick, H. *et al.* Differential brain activity in alcoholics and social drinkers to alcohol cues: relationship to craving. *Neuropsychopharmacology* **29**, 393-402, doi:10.1038/sj.npp.1300295 (2004).

76 Potenza, M. N. *et al.* Neural correlates of stress-induced and cue-induced drug craving: influences of sex and cocaine dependence. *Am J Psychiatry* **169**, 406-414, doi:10.1176/appi.ajp.2011.11020289 (2012).

77 Ray, S., Haney, M., Hanson, C., Biswal, B. & Hanson, S. J. Modeling Causal Relationship Between Brain Regions Within the Drug-Cue Processing Network in Chronic Cocaine Smokers. *Neuropsychopharmacology* **40**, 2960-2968, doi:10.1038/npp.2015.150 (2015).

78 Tang, D. W. *et al.* Genetic variation in CYP2A6 predicts neural reactivity to smoking cues as measured using fMRI. *Neuroimage* **60**, 2136-2143, doi:10.1016/j.neuroimage.2012.01.119 (2012).

79 Tomasi, D. *et al.* Overlapping patterns of brain activation to food and cocaine cues in cocaine abusers: association to striatal D2/D3 receptors. *Hum Brain Mapp* **36**, 120-136, doi:10.1002/hbm.22617 (2015).

80 Mitchell, M., Baurzhan, M. & Tobias, W.

81 Volkow, N. D. *et al.* Cognitive control of drug craving inhibits brain reward regions in cocaine abusers. *Neuroimage* **49**, 2536-2543, doi:10.1016/j.neuroimage.2009.10.088 (2010).

82 Vollstädt-Klein, S. *et al.* Initial, habitual and compulsive alcohol use is characterized by a shift of cue processing from ventral to dorsal striatum. *Addiction* **105**, 1741-1749, doi:10.1111/j.1360-0443.2010.03022.x (2010).

83 Vollstädt-Klein, S. *et al.* Severity of dependence modulates smokers' neuronal cue reactivity and cigarette craving elicited by tobacco advertisement. *Addict Biol* **16**, 166-175, doi:10.1111/j.1369-1600.2010.00207.x (2011).

84 Wang, A. L. *et al.* Neural correlates of adherence to extended-release naltrexone pharmacotherapy in heroin dependence. *Transl Psychiatry* **5**, e531, doi:10.1038/tp.2015.20 (2015).

85 Wrase, J. *et al.* Dysfunction of reward processing correlates with alcohol craving in detoxified alcoholics. *Neuroimage* **35**, 787-794, doi:10.1016/j.neuroimage.2006.11.043 (2007).

86 Xiao, Z. *et al.* Thirsty heroin addicts show different fMRI activations when exposed to water-related and drug-related cues. *Drug Alcohol Depend* **83**, 157-162, doi:10.1016/j.drugalcdep.2005.11.012 (2006).

87 Yalachkov, Y., Kaiser, J. & Naumer, M. J. Brain regions related to tool use and action knowledge reflect nicotine dependence. *J Neurosci* **29**, 4922-4929, doi:10.1523/JNEUROSCI.4891-08.2009 (2009).

88 Zanchi, D. *et al.* Cigarette smoking leads to persistent and dose-dependent alterations of brain activity and connectivity in anterior insula and anterior cingulate. *Addict Biol* **20**, 1033-1041, doi:10.1111/adb.12292 (2015).

89 Zeng, H. *et al.* The Action Representation Elicited by Different Types of Drug-Related Cues in Heroin-Abstinent Individuals. *Front Behav Neurosci* **12**, 123, doi:10.3389/fnbeh.2018.00123 (2018).

90 Zijlstra, F., Veltman, D. J., Booij, J., van den Brink, W. & Franken, I. H. Neurobiological substrates of cue-elicited craving and anhedonia in recently abstinent opioid-dependent males. *Drug Alcohol Depend* **99**, 183-192, doi:10.1016/j.drugalcdep.2008.07.012 (2009).

1. One study^27^ involved comorbid cocaine- and heroin-dependent individuals, involved in both heroin- and cocaine-related mental imagery. [↑](#footnote-ref-1)
